# Time Series Analysis for Physiological and Endocrinological Data: A Practical Guide

**DOI:** 10.1093/icb/icag092

**Published:** 2026-06-18

**Authors:** Joshua Reed, Cory J D Matthews, Jennifer Jelincic, C Loren Buck, Kathleen E Hunt, Leslie New

**Affiliations:** Department of Mathematics, Computer Science and Statistics, Ursinus College, Collegeville, PA 19426, USA; Fisheries and Oceans Canada, 501 University Crescent, Winnipeg, MB R3T 2N6, Canada; Marine Mammal Institute, Oregon State University, Newport, OR 97365, USA; Department of Community, Environment and Policy, Mel & Enid Zuckerman College of Public Health, University of Arizona, Tucson, AZ 85724, USA; Marine Mammal Institute, Oregon State University, Newport, OR 97365, USA; Department of Mathematics, Computer Science and Statistics, Ursinus College, Collegeville, PA 19426, USA

## Abstract

The study of endocrinology provides insights into the upstream drivers of behavior and physiology of wild and captive populations to both natural and anthropogenic stressors. Most studies of wildlife endocrinology rely on single samples from multiple individuals to understand differences between different demographic states or populations. However, neither stressors nor hormone secretion is static, fluctuating over time within and between individuals. The use of nontraditional sample types that accumulate hormone concentrations through time, or repeated sampling of individuals through time can elucidate natural hormone fluctuations and the influence of stressors over time. With studies containing a temporal component come numerous challenges when interpreting and analyzing the data due to the inherent correlation between data points. Time series analysis is a group of statistical methods that were developed to analyze data collected with a temporal component. Here, we present a review of classic time series analysis methods, describing how they can be used with endocrinology data while providing worked examples and R code. By integrating time series analysis into endocrinology studies, researchers can get a better understanding of the temporal and individual variation in specific hormones. Through highlighting these tools and how they can be applied, we hope that they can be more readily available to all members of the endocrinology field to further understand wildlife endocrinology.

## Introduction

Endocrine markers can provide insights into the responses of wildlife to natural and anthropogenic stressors, and inform our understanding of demographics, reproduction, and nutrition, often using less invasive techniques (Kersey and Dehnhard 2014). To set realistic conservation objectives, it is important to understand long-term changes in individual endocrine states across time (Hudson et al. 2021). Importantly, hormones (and other physiological biomarkers) can be quantified in a wide variety of vertebrate sample types that can capture different timeframes (Romero and Wingfield 2015), e.g. minutes for blood (McKenzie et al. 2004) and respiratory vapor (Burgess et al. 2018), hours for urine (Lasley and Kirkpatrick 1991) and blubber biopsies (Kershaw and Hall 2016), days for feces (Rolland et al. 2012), and weeks to years for slow-growing tissues such as skin epidermis (Bechshoft et al. 2015; Zena et al. 2022), spines (Fabio Braga et al. 2022), hair (Calamari et al. 2020), and feathers (Adámková et al. 2019). Yet sampling a target individual repeatedly over time can be difficult or near impossible, especially for free-ranging species (Hunt et al. 2006).

Over the last three decades, advancements have been made with certain linearly growing sample types, such as whiskers (Keogh et al. 2021), teeth (Hudson et al. 2021) and baleen (Hunt et al. 2014, 2026) being found to accumulate hormones in sequential fashion along the length of the tissue (or across nested annual growth increments, e.g., teeth), thus creating a time series of biomarker data arrayed along a single physical specimen. Such tissues have allowed for the exploration and study of longitudinal hormone profiles of individual free-ranging mammals (Hunt et al. 2016, 2018; Keogh et al. 2020; Crain et al. 2021; Keogh et al. 2021). Often these time-series hormone datasets are combined with analysis of stable isotope ratios to understand the timeframe represented by the tissue, as well as investigate foraging ecology, movement, niche partitioning, and how these factors could influence circulating concentrations of specific hormones such as cortisol (Burstahler et al. 2019; Karpovich et al. 2019; Crain et al. 2021), corticosterone (Barger and Kitaysky 2012), and progesterone (Crain et al. 2021). Stable isotopes thus provide additional information on individuals’ nutritional and physiological state, as well as clarifying temporal context (e.g., identification of specific calendar years of growth along the specimen) (Schell et al. 1989; Rea et al. 2015; Fleming et al. 2018) and seasonality (Matthews and Ferguson 2015a; Hunt et al. 2018; Lysiak et al. 2018; Pomerleau et al. 2018). These longitudinal datasets therefore require analysis of temporal patterns such as cycles, seasonal effects, and trends over time, yet many wildlife endocrine studies do not take full advantage of the many statistical techniques that have been developed specifically for such datasets.

Time series data are common in many fields, including economics (stock prices, interest rates), meteorology (temperature, rainfall), and biology (heart rates, neural activity, and population abundance), consisting of data that are collected on the same observational unit periodically over time (Shumway and Stoffer 2006). Due, in part, to the dependent nature of the data (e.g., each data point is typically influenced by its neighbors), time series analysis poses unique problems that restrict using many common statistical methods. To overcome these challenges, various mathematical and statistical models have been developed which aim to understand the stochastic mechanisms driving the observed time series, and to predict future values of a series (Cryer and Chan 2008). These models, although developed for data very disparate from endocrine profiles, are fully applicable to the study of the change of hormone concentrations over time (Matthews 1988). As a result, while the nature of the biomarkers and matrices that give rise to the time series hormone data sets vary, all can be analyzed with similar approaches.

Many time series contain patterns that, with appropriate modelling and interpretation, can facilitate insights into the underlying dynamics of the study system. Classical time series analyses often assume that measurements are taken from the observational unit at known periods of time, such as clinical studies in which blood samples are collected at known timepoints to investigate the hormone profiles of patients over time (Vis et al. 2012). However, many recent studies on slow-growing tissue types face the additional challenge that the timeline is not initially known, i.e. the timepoint represented by each location along the specimen must be inferred from other information such as species and tissue-specific growth rate, annual shifts in foraging ecology, and a known anchor point such as date of death or sample collection, which adds complexity to the analyses. Stable Isotopes provide a useful tool understanding the movement of migratory animals which can be used to identify the location the tissue was grown in (Hobson 1999) which can then be used with known timings of migratory events for that species to estimate time. For example, oxygen stable isotope (δ^18^O) concentrations were originally analyzed from barnacle plates living on gray whales to understand migration (Killingley 1980). By sampling the growth layers of the barnacles, a time series of oxygen concentrations revealed the location of where that segment was grown.

To date, statistical methods for applications of time-series analysis to wildlife endocrinology have not been published since the late 1980s (Clifton and Steiner 1983; Matthews 1988), yet there has been substantial development in the field, both statistically and computationally, since that time (Shumway and Stoffer 2006). In this review paper, we seek to provide an overview and a primer on modern time-series analytic techniques useful for endocrinological (and, more generally, physiological), time-series data. We focus on applications to marine mammal endocrinology specifically, due to the recently burgeoning number of time-series datasets in this field, but the techniques discussed herein are applicable to other taxa as well. Our goal is to provide a single resource that highlights the statistical methods applied by the community, as well as providing R code (R Core Team 2024) (with all analyses conducted in R) and other information to make time series analysis more readily available to individuals unfamiliar with these methods. This information has been made available as a series of *Supplemental Information* (*Supplemental Information—code; model details; resources;* and *further worked examples*). Finally, we discuss directions beyond the standard models, to highlight the scope of time series analyses applications in endocrinology.

### Guide to this resource

**Fig. 1:**
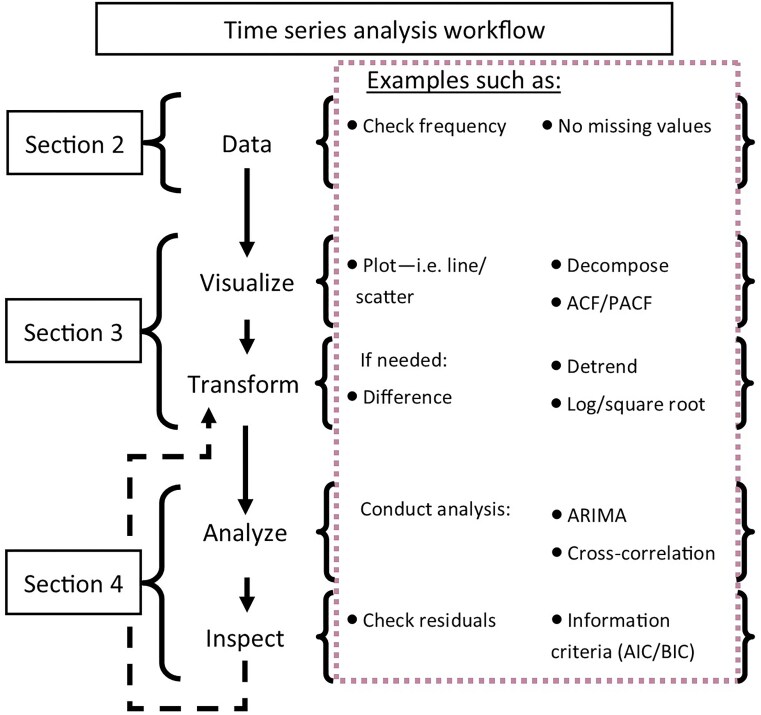
A suggested workflow for time series analysis. The steps are outlined in the center with solid arrows indicating the workflow order. Steps are divided into sections, indicating the corresponding sections of the paper where they can be found. The dotted line indicates the path if the analysis does not work. The parentheses next to each step provide some examples of what these could look like for each step, however, there are many more options available for each step.

To make this resource as user friendly as possible, we have divided the paper into three main sections. First, we start off with longitudinal data and sampling design, followed by the structural components and assumptions of time series analysis, and finally we present common methods for time series analysis that are suitable for endocrinology and physiological data. Our suggested workflow for conducting time series analysis is outlined in Fig. 1.

## What is Longitudinal Data?

Time series consist of data (${y_t}\,\,:t = 1,.\,\,.\,\,.,\,\,T$) collected from an observational unit through time; here the subscript *t* represents the time at which the data was collected or observed, while *T* represents the final time point in the series. Time series data are inherently *autocorrelated*, meaning current values at time *t* are influenced by previous values in the series *t_-T_* (Bartlett 1946). Each data point in the series can represent a single point (i.e., a blood sample representing concentrations at the time taken) or an accumulation of an underlying quantity over an interval of time (i.e., baleen samples where concentrations at a single point on the specimen likely represent multiple days of circulating hormone). The majority of commonly applied time series analysis approaches require data collected at equally spaced intervals, but there are methods that relax this assumption and allow for multiple series, i.e. different analytes, such as multiple hormone or stable isotope concentrations from the same sample (Diggle and Giorgi 2025). Further, the resolution of the sample type can vary from seconds to years, depending on the tissue type, the method by which circulating hormone enters the tissue (e.g., deposited via bile into feces, or diffusing from an epithelial growth zone into keratin), as well as the tissue growth rate, the consistency of that growth rate (e.g., feathers only grow during certain months of the year), and, for hard-part tissues, the spatial sampling interval along the sample (Sheriff et al. 2011; Hunt et al. 2016; Adámková et al. 2019; Calamari et al. 2020; Keogh et al. 2021). This myriad of temporal factors must be considered carefully for each tissue type and each species, as they will influence the types of research questions that can be addressed.

### Sampling design and considerations

#### Sampling intervals

Choosing the right sampling design to answer longitudinal questions is important to ensure that the correct temporal resolution can be obtained. For linearly grown keratinous tissues, analytes can be sampled along the length of the sample, with the sampling interval determined by the growth characteristics of the specific tissue.

Tissues with discrete growth layers (e.g., horn, tusk, or tooth) offer a clear temporal guide, as each growth layer represents a specific growth period as the temporal resolution is inherently structured by the biology of the tissue (Fisher et al. 2014 ; Hudson et al. 2021; Cherney et al. 2023).

For tissues that lack discrete growth layers (e.g., vibrissae, baleen), determining an appropriate sampling interval first requires establishing the growth rate of the tissue. Species-specific growth rates can be estimated using stable isotope analysis, though age-class-specific growth rates can also be estimated, where isotopic shifts along the length of the tissue provide a time-equivalent record of dietary and/or habitat change. This then provides a tissue length that is functionally equivalent to a unit of time and can be used to help determine the sampling interval (Matthews and Ferguson 2015a; Rea et al. 2015; Keogh et al. 2021).

Unlike keratinous tissues, nonkeratinous biological matrices (e.g., blood, plasma, feces), reflect brief windows of analyte concentrations and require repeated sampling of individuals. Repeated sampling allows for greater control and temporal resolution of the data, such as collecting blood every hour to look at finer scale questions.

#### Number of Samples

Many of the common time series analysis methods call for > 50 samples to reliably describe the data, and > 2 seasonal cycles if looking at seasonality. However, this can often be an oversimplification, as it ignores the underlying variability of the data (Hyndman and Kostenko 2007). From a statistical view, a model needs more observations than parameters as an absolute minimum, e.g. if we fit an autoregressive AR(1) model we would have three parameters: (1) the AR coefficient, (2) the intercept or constant, and (3) the variance; meaning we would need at least four samples for this analysis (see section Autoregressive) This, however, does not consider randomness in the data, with hormone data known to be inherently noisy due to biological and methodological factors (Fanson and Fanson 2015). When the data are increasingly noisy, the number of samples needed will increase in order to accurately estimate the model (Hyndman and Kostenko 2007). There are no set rules for how many samples will improve model estimation. It can be considered instead in terms of margins for error. The minimum samples required provides you with large error, while if the sample size is increased the error decreases proportional to the square root of the sample size (i.e., if you quadrupled the number of samples, the error would be halved) (Hyndman and Kostenko 2007). Large sample size is particularly important for models where forecasting beyond the data are a primary outcome.

#### Limitations

An important practical consideration when working with continuously grown tissues is ensuring that the sampled sections (i.e., growth layers or tissue segments) contain sufficient material to measure analyte concentration (whether for hormone assay, stable isotope analysis, or both). Because the length or layer of tissue representing a biologically meaningful unit of time (e.g., one month of growth) may only result in a small amount of material. Therefore, the length of segments or pooled growth layers may need to be increased to obtain sufficient weight for reliable analysis. This introduces a trade-off: longer segments provide greater mass and analytical sensitivity, but at the cost of temporal resolution. Conversely, shorter segments preserve finer temporal resolution but risk falling below the minimum detection threshold for assays. The optimal balance between segment length (temporal resolution) and segment mass (analytical sensitivity) will therefore vary depending on the tissue type, growth rate, number of analytes, and the sensitivity of the assay being used. We recommend conducting a preliminary study to test this balance before committing to a full sampling design. For nonkeratinous matrices, there are significant limitations with being able to sample the same individual multiple times at pre-determined time intervals, particularly for wildlife studies.

### Assumptions of data for time series analysis

As we have seen, there are a number of considerations for sampling endocrinological and physiological data. However, there are also considerations that need to be made when considering the right analysis for your data, and that your data are ready to be modeled.

Most time series analyses have two key assumptions:

1. Samples are evenly spaced—That the data are systematically sampled at equal time points, i.e. daily. This assumption is the foundation of many classical time series methods in order to accurately calculate trends, lags, and seasonality from the constant relationship between sequential time steps. When working with nonkeratinous sample matrices, such as bloods, the spacing of the samples can be predetermined that reflect the window of the sample matrix, such as one sample every 10 mins over a day for blood, or sampling every day for a month for feces. When dealing with keratinous tissues, we don’t know the exact temporal resolution of the underlying time sequence and thus use measured distance along the sample as a proxy for time. However, these tissues do not necessarily grow at a constant rate throughout an animals life and the same distance in one section of tissue could represent a different temporal resolution to another. We can assume that the data are easily spaced to conduct analysis, though our results are likely to be biased due to this. There are methods available for unequally spaced samples (Broersen and Bos 2006), however, these also generally require the spacing of the intervals between samples to be known. Grouping samples in to bins of averages is another way in which unequally spaced samples can be used in time series models.
2. Data are stationary—Another key assumption is that the data used is stationary, meaning there is no trend and equal variance within the time series (Cryer and Chan 2008). There are a number of ways to ensure that the data meet this assumption, differencing and detrending (see section Trend) can help remove trends from the data and can aid to normalize variance. For variance, transforming the data using a Log or Box-Cox transformation often further aid to stabilize the variance. By modelling nonstationary data, the model results will likely be biased with underestimated standard errors. By accounting for these in the model by either making the data stationary or using a model that can handle them (see section Autoregressive Conditional Heteroskedasticity models) we can better explore the structure of the time series, and then methods such as amplitude analysis (section Amplitude analysis) can be used to look at changing variance or change point models (section Change point models) can be used to look at changing means in the time series data.

## Decomposing key structural elements of longitudinal data: trend, seasonality, and noise

**Fig. 2:**
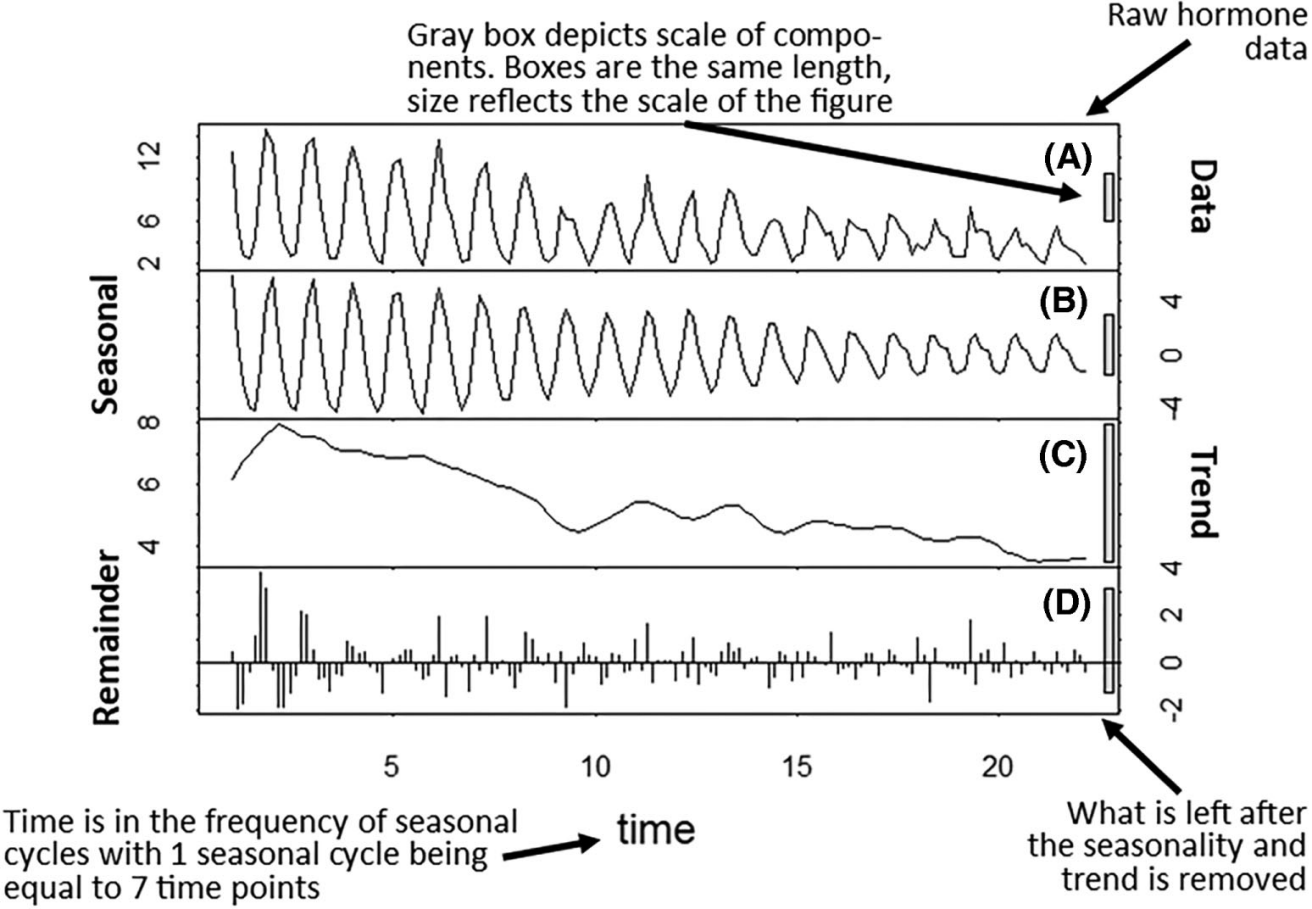
Time series components of the Male bowhead testosterone dataset. (A) shows the raw data, (B) the seasonal component, (C) the trend in the data, and (D) the residuals which are the components that cannot be explained by the trend or seasonal components.

Time series analysis was designed to develop mathematical and statistical models to describe sample data that fluctuates with time. A time series can be defined as a collection of random variables that are ordered, or indexed, in the order in which they are collected (Kirchgässner et al. 2012). Consider a time series of testosterone concentrations (Box. 1, Fig. 2) from baleen sub-samples collected incrementally from an individual baleen plate, ${y_1},\,\,{y_2},\,\,{y_3},\,\,.\,\,.\,\,.,\,\,{y_t},$ where ${y_1}$ is the sample collected from the first incremental sample location, ${y_2}$ the sample from the second incremental sample location, and so on up to the total number of incremental samples from the length of the baleen plate. Time series data $[ y ]$, such as our hormone data, represent the evolution of a situation over time, and are thus what is known as a stochastic process. Time series can be broken into their key components: trend (T_t_); seasonality (S_t_); and the remainder (R_t_) (Hyndman and Athanasopoulos 2018):


\begin{eqnarray*}
{y_t} = \,\,{T_t} + \,\,{S_t} + \,\,{R_t}
\end{eqnarray*}

Such a dataset is often best initially visualized via a scatterplot with time along the x-axis and the dependent variable, in our case hormone concentration, on the y-axis. This allows for a preliminary determination as to whether there may be (1) a trend in the data; (2) a change in the variance of the data with respect to the mean; and/or (3) seasonality (repeated cycles in the data of approximately equal length). Time series analysis generally requires time series to be stationary (no trend or variance), and seasonality accounted for, which will be described in more detail below. While failure to observe any of these patterns is not evidence of their absence (e.g., a noisy process may obscure patterns), their visual presence is a quick indicator of the need to account for them in the analysis.

### Trend

The trend in a time series refers to the long-term direction or movement in the data, regardless of any seasonal or cyclical fluctuations (Ogasawara et al. 2025). Trend describes the upward and downward movements over the entire time series; lack of trend indicates no long-term movement; capturing the underlying, smooth changes in the time series which can be linear, exponential, nonlinear or other patterns. For example, testosterone concentrations in a juvenile male of a species may increase over time as they come into sexual maturity, a positive trend. Estrogen and progesterone in adult females may decline throughout life as individuals go through menopause or senescence, a negative trend. Characterizing trends help us understand how different hormones can change through time and for different demographic groups. If there is a trend detected in the time series, this is referred to as nonstationary data. While trends may be of interest in and of themselves and should be looked at to understand what is happening with trend, however, for any other time series analyses the trend then needs to be removed (see Box 1) or accounted for in the analysis (more details in models below).

Removing a trend entails a process known as detrending, which is performed in order to focus on the time series components that could otherwise be masked by the long-term direction of movement in the data. Many time series analysis methods have an underlying assumption that the data are stationary with no trend, as having a trend in the data could inflate the correlation between timepoints, however, other models are available that can explicitly account for the trend in a time series. Detrending can be done in several ways (Shumway and Stoffer 2006), with two common approaches being (1) linear regression, where if the trend is linear, the predicted trend from the model can be subtracted from the original data; and (2) differencing, where the value of the current time step is subtracted from the previous time step in its simplest form. By removing the trend or making the data stationary we meet one of the assumptions of most time series analyses, no variance or trend in the data. Worked examples of these two methods can be found in the *Supplemental Information- code*.

### Seasonality

Seasonality in a time series refers to cycles that repeat throughout the data at fixed intervals (i.e., daily, weekly, monthly, or yearly). This is known as fixed periodicity. Cycles are characterized by having a period, the specific position of the cycle in time, and an amplitude, the magnitude of the fluctuations from the long-term trend. The specific amplitude of the cycles may vary (Ogasawara et al. 2025). Period and amplitude help us understand the temporal nature of the cycle, as well as the magnitude increase in hormone concentrations above baseline values. Seasonal cycles (i.e., annual, monthly or daily cycles in which hormone rises and falls during a period of time) usually relate to periodical mechanisms which underlie the data, e.g. testosterone in mature males typically increase during the breeding season and decrease in the nonbreeding season (Hunt et al. 2022). Understanding seasonal patterns in a specific hormone (or accompanying stable isotope values) can thus help identify life history events such as reproduction, migration, or interbirth intervals.

Analysis of time series data needs to account for identified seasonality, just as with trends more broadly, as such cycles can mask other underlying patterns in the data. However, the seasonality may be the component of the time series that is of interest such as with many stable isotope studies (Matthews and Ferguson 2015a; Pomerleau et al. 2018; Hunt et al. 2022), in which case the seasonal component can be kept to model this seasonality. By removing the seasonal component from your data, insights can be gained about how the data are temporally correlated without the known seasonal patterns, however, for biological systems, including the seasonal patterns is often favorable to understand the mechanisms that could be driving changes. Common methods to account for seasonality include (1) seasonal differencing, where the value of the current time step is subtracted from the same point in the previous season (i.e., *t* − 12 for a yearly cycle, *t* − 3 for a quarterly cycle) (*Supplemental Material—code*); and (2) seasonal models, which directly account for seasonality in the analysis to estimate the seasonal effect while separating it from the underlying trends (more details are provided in specific models below).

### Remainder

The remainder represents the random fluctuations within the time series that are not accounted for by the trend or seasonal components (Ogasawara et al. 2025). This is often referred to as “noise” in time series analysis, but such noise may not be random, and may reflect biological patterns of interest. For example, we may want to focus on the remainder to assess how glucocorticoid hormone concentration varies in a captive animal to capture irregular events such as changes to enclosures, without the influence of known patterns such as seasonal fluctuations in stress due to changes in diet, and a trend due to age.

Box 1. Time series components

Here we present how to identify and correct for trends and seasonality within time series data, using a dataset of *male bowhead testosterone*. Visual assessment of the time series indicates a potential trend given the increase in the data value in the middle of the data set. In addition, there is a recurring pattern of three peaks followed by an extreme minimum indicating the presence of cycles. We can also more clearly break the time series down into its components, trend, seasonality and the remainder using the *stl* function in R (R Core Team 2024) (Fig. 2). Doing so further highlights the presence of the trend (Fig. 2C) and the strong seasonal component to the data (Fig. 2B).

**Fig. 3:**
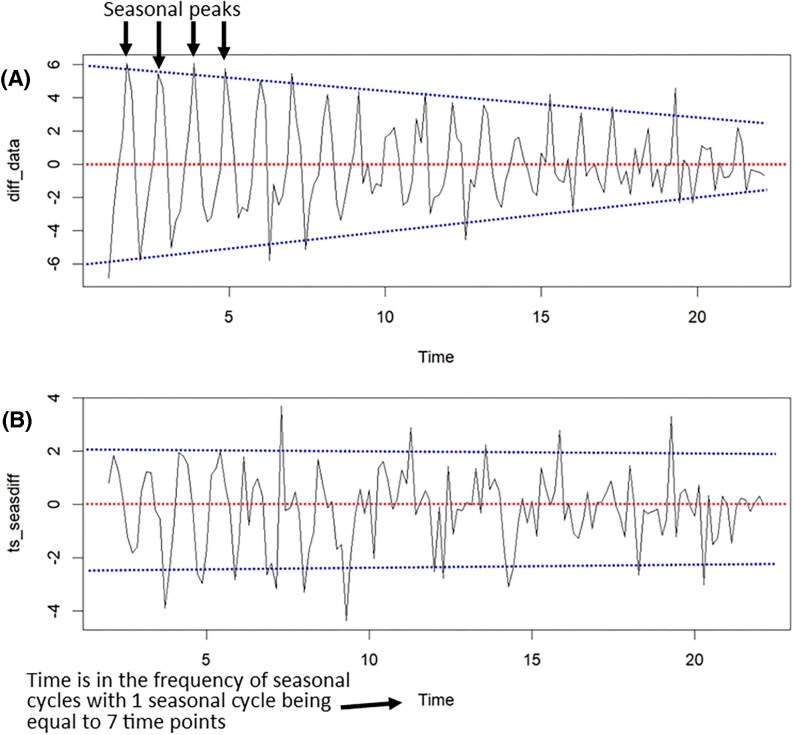
The detrended data for the testosterone data with (A) regular detrended and (B) seasonal detrended. The dotted line at zero shows the centered mean; the upper and lower dotted lines show the rough spread of the variance.

To use these data in more in-depth time series analysis, it is necessary to account for trend and seasonality. In this case, we accounted for trend by detrending (Fig. 3A), and seasonality through seasonal differencing (Fig. 3B), although seasonality can also be accounted for in the analysis where applicable (see below for more details).

In the detrended data (Fig. 3), we can see that we still have strong seasonal peaks in the regular detrended data (Fig. 3A), while those peaks are reduced to account for the seasonality in the seasonal detrended data (Fig. 3B). This data set is now ready for use in our time series analysis. Code for recreating these steps is available in the *Supplemental Information - code*.

## Time series analyses examples for endocrinology and physiology

Time series data are common in numerous fields, and there have been many different methods developed to understand and interpret these data. However, the classical time series models remain the most frequently used, especially within endocrinology. This is due to their simplicity, ease of interpretation, and efficiency. It is important to note that for these methods, they are applied to a single time series, meaning if there are multiple individuals/analytes per individual, the analyses will need to be conducted separately for each. Here, we present exemplar classical time series analyses that provide the foundation for these methods within endocrinology studies, illustrated with typical case studies using hormone and stable isotope data measured at 2-cm increments along baleen plates of adult male bowhead baleen published in Hunt et al. (2022). We use baleen as the example throughout this paper for consistency for the readers.

### ARIMA models

Regression is often insufficient in explaining the nuances of time series data, failing to capture the intercorrelated structure of the data (Shumway and Stoffer 2006). This led to the development of AR and autoregressive moving average (ARMA) models, which account for the correlation between lagged linear relationships (Whittle 1951). These methods were later revised to account for nonstationary data (data with a trend) with the autoregressive integrated moving average (ARIMA) model (Box and Jenkins 1976). The models can be broken down into three components: the AR, moving average (MA), and the integrated (I).

#### Autoregressive

The AR component indicates the regression between present values in the time series, ${y_t}$, and prior values of the same time series, ${y_{t - 1}},{y_{t - 2}},{y_{t - 3}},.\,\,.\,\,.,\,\,{y_{t - p}}$. This is based upon the idea that current values of the time series can be explained as a function of *p* past values, where *p* is the number of past time steps (lags) needed to predict the current value, and is known as the model order. To determine the number of lags required to predict current values in the time series, one can use a *partial autocorrelation* function (PACF). The PACF is a conditional correlation, meaning the correlation between two variables is influenced by the values of other variables for which we have data. Partial autocorrelation is a value between 0 and 1 with values closer to one indicating strong partial autocorrelation, while values closer to zero indicate weak to no partial autocorrelation. The number of values, or lags, that are identified as statistically significant indicate the model order, i.e. the number of lags correlated with the value ${y_t}$. The model order is set to the maximum lag (the number of significant lags) to ensure the underlying structure of the data is represented without overfitting the model. Metrics such as Akaike Information Criterion (AIC) can be used to test models from 1: max lag to find the best fitting model that balances too many parameters with an informative model. More information on the statistical model details of AR models can be found in the *Supplemental Information—Model details*. In endocrinology, AR models can be used to explore seasonality in hormone and stable isotope concentrations (Matthews and Ferguson 2015a), to understand how many past time steps influence the current values of a given hormone to help understand temporal lag, as well as a tool for accounting for temporal correlation to be used with other analyses such as spectral analysis (see *Spectral analysis* for more information). Outside of describing the existing series, AR models can be used to predict future values of the time series using past values. A practical endocrinological application, e.g. could be predicting future contraceptive timings of individuals in captive breeding programs. Box 2 gives an example of fitting the steps of fitting an AR model and the PACF.

Box 2. Autoregressive

**Fig. 4:**
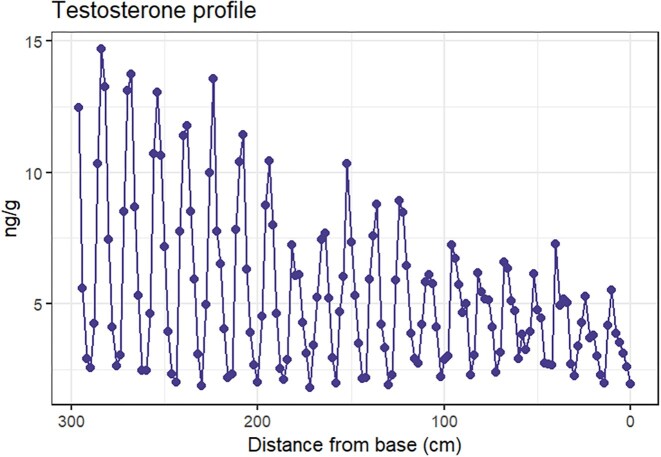
Testosterone profile for male bowhead whale with concentration in ng/g on the y-axis, and sample location as distance from the base in cm on the x-axis.

Here we present how to conduct an AR model using the *male bowhead testosterone data* (Fig. 4). The first step is to detrend to remove the trend from the data, we did this through differencing using the *diff* function from the ‘stats’ function in R (R Core Team 2024). We set the number of differences as 1. If you are interested in removing the seasonal component, you can also seasonally difference in this function using the ‘lag’ argument. Differencing can also help stabilize the variance, and the data can be plotted to check and transformed if there is still heteroscedasticity.

**Fig. 5:**
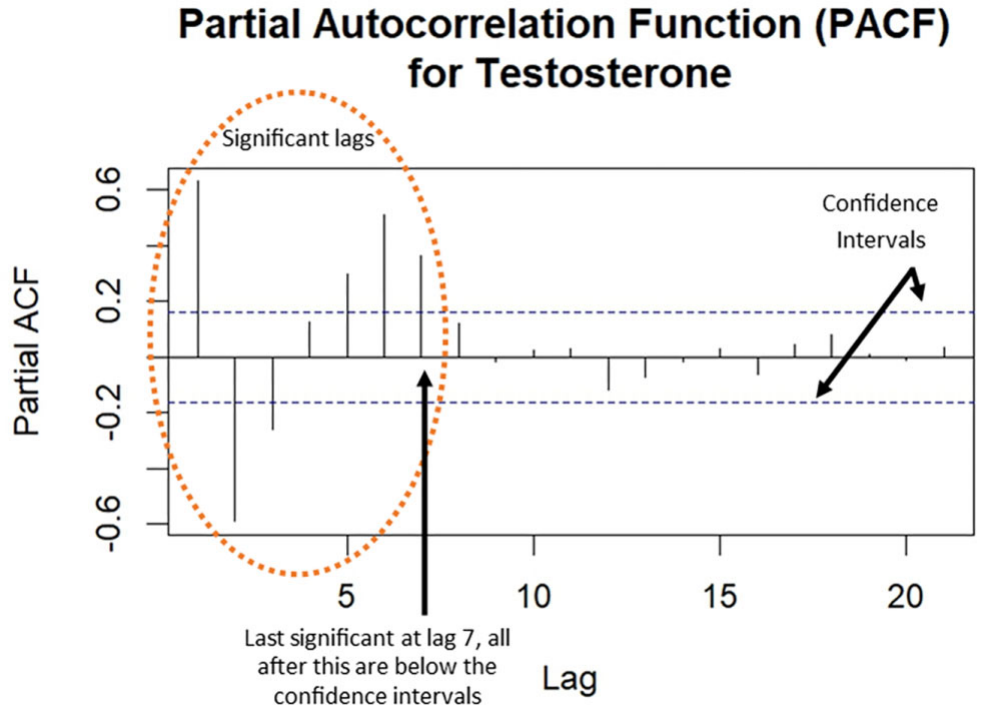
The partial autocorrelation function for the testosterone profile, with lags on the x-axis and correlation on the y-axis. The horizontal dotted lines indicate the confidence intervals, with values over the line indicating significant lags, indicated by the orange circle.

Once the dataset is stationary, we can fit the PACF using the *pacf* function from the “tseries” package (Trapletti and Hornik 1999) in R. The PACF will produce a plot (Fig. 5) where spikes that cross the blue-dashed lines indicate autocorrelation values that are statistically significant. The highest lag with a significant spike can be used as your model order in the autoregressive model. The significant lags indicate that there is autocorrelation between these points.

Using the model order we can now fit the autoregressive model using the *ar* function from the “stats” package in R, where we set “order.max” equal to our model order (in this case 7). Model fitting such as AIC can then be used to compare models between 1-order max to find the appropriate model structure. The output of the AR model can then be used to predict future values using the *forecast* function from the ‘forecast’ package (Hyndman and Khandakar 2008) in R, or used in conjunction with other analyses to address specific questions with the predicted data.

#### Moving average

The MA component assumes that ${y_t}$ is linearly dependent on the current and previous noise in the data (i.e., the error term). This model differs from the AR model in that it does not use past values of the time series to understand the structure of the time series, but rather the past error. By accounting for the uncertainty in previous time steps, MA models smooth out irregularities in the data and provide insights into underlying trends. Similar to the AR model, the MA model is determined by the number of lags that are autocorrelated with ${y_t}$. To do this, we use the *autocorrelation* function (ACF) (not to be confused with the PACF function used in the AR model). Like the partial autocorrelation, autocorrelation is a value from 0 to 1, with values closer to one indicating a strong autocorrelation, while values close to zero indicate weak to no autocorrelation. As before, the number of lags identified as statistically significant indicate the model order, which is now identified with *q* to indicate it is the order of an MA model. More information on the statistical model can be found in the *Supplemental Information—Model details*. MA models and ACF functions are useful for looking for seasonal patterns in hormone data (Hudson et al. 2025), and as an initial step to characterize variation in data with an MA process for further analyses. Similar to the AR model, the results of the MA model can be used to predict future values of the time series. *SI Box.1* in the *Supplemental Information—further worked examples* provides an example of fitting the steps of an MA model and the ACF using real data.

#### Autoregressive moving average

ARMA models combine both the AR and MA components into a single model that can combine both the past errors and values of the time series to help understand the structure of the data. As with the AR and MA models, the PACF and ACF functions need to be used to understand the number of previous time periods (lags) needed to predict the current value. The ARMA model assumes that the data are stationary, however, advancements to the ARMA model with the inclusion of an integrated component allow for differencing to occur during the model in the ARIMA model.

#### Autoregressive integrated moving average

The ARIMA model is often considered the most general time series model. It combines components of the AR and MA models, while adding in an integrated component, which uses differencing to ensure the time series is stationary. In doing so, the trend is removed from the time series enabling focus on the underlying structure of the data (see Time series models above for more details on trends in time series data). The ARIMA models aim to separate the signal in the time series from the noise, which can then be extrapolated to forecast future values of the series. Further details on the statistical model can be found in the *Supplemental Information—Model details*. Similar to the AR and MA models, the first steps of ARIMA models require estimating partial autocorrelation and autocorrelation to determine the number of lags to include in the model. The new step is the differencing, which is applied until the time series is stationary or together with another form of transformation (e.g., taking the natural log of the data to stabilize the variance). Once the amount of differencing, and number of lags have been calculated, the ARIMA model can be fit. Higher-order ARIMA models (i.e., a large number of lags) have been used to calculate period length in baleen (Matthews and Ferguson 2015a; Pomerleau et al. 2018). However, it is important to note that higher order models can encounter over-fitting issues and thus require more computational time. *SI Box 2* in the *Supplemental Information—further worked examples* provides more details for fitting an ARIMA model.

##### Seasonal ARIMA

Seasonality in the data can also be accounted for within the ARIMA framework. Incorporating seasonal trends into the ARIMA model follows the same structure as the regular ARIMA model, now identifying those factors that occur across multiple lags of *s*, the number of periods occurring within a season (i.e., 12 months for yearly trends).

Determining if seasonal differencing is required can be done by looking at the plotted time series data, in combination with the ACF and PACF plots. If the seasonal pattern appears strong and consistent over the time series, then a seasonal difference should be used. For seasonal data, seasonal differencing should only be used once, and there should not be more than two total differencing (i.e., one seasonal and one nonseasonal differencing). If differencing is required, the next step is to determine whether AR or MA components are also required. The patterns of the seasonal ACF and PACF will now appear across multiple lags at the seasonal peaks (i.e., every 12 points for data collected monthly across multiple years). Using more than two total differencing can lead to over differencing which can introduce noise into the data and decay the structure of the underlying data (Kumar and Vanajakshi 2015). A seasonal AR component can usually be identified by a positive autocorrelation at the seasonal periods, while a negative autocorrelation usually suggests a seasonal MA component. It is therefore suggested that a seasonal ARIMA model only includes an AR or MA component and not both.

### Cross-correlation

Understanding the relationship between two variables can provide important information about how they influence each other, e.g. how corticosterone (indicative of physiological stress) covaries with testosterone (mating season) or δ^15^N (migratory events) (Hudson et al. 2025). Such relationships can involve time lags of biological interest, e.g. the classic effect of prolonged stress on subsequent reproduction (e.g., unusually high corticosterone in one cycle followed by unusually low progesterone or testosterone in the next). In endocrinology the interactions between different variables can provide invaluable information on how two hormones interact, or how external factors such as temperature, prey availability, or disturbance level can influence certain hormones. Cross-correlation is a useful time series model that measures the relationship between two time series as a function of the relative lag between them (Cryer and Chan 2008). When you have two time series (${y_t}$ and ${x_t}$), values of the ${y_t}$ series could be related to past values of the ${x_t}$ series, meaning that lags (past values) of ${x_t}$ could be useful in predicting present values of ${y_t}$. Cross-correlation works by assessing the correlation between ${x_{t + h}}$ and ${y_t}$ where $h = 0,\,\, \pm 1,\,\, \pm 2,\,\, \pm 3,.\,\,.\,\,.$ up to the maximum number of lags. A positive value for *h* represents a correlation between a future value of a variable and ${y_t}$, while a negative value of *h* is a correlation between a past value of a variable and ${y_t}$. When a variable with a positive *h* is correlated with ${y_t}$, it can be said that *x* lags *y*, while when negative *h* values are correlated with ${y_t}$, *x* leads *y*. Identifying which time series variable is leading and which is lagging helps us understand how they are interacting. Cross-correlation is often a useful first step in understanding the temporal correlation between two variables and can lead to more involved time series analyses such as multivariate analysis (See *Purpose built models* below).

Box 3. Cross-correlation

**Fig. 6:**
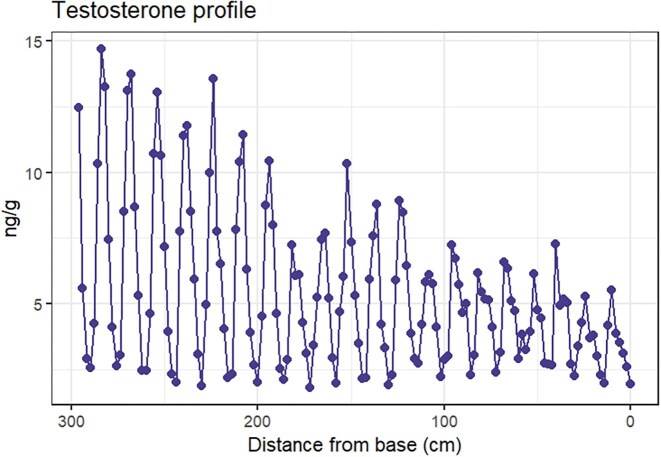
Testosterone profile, with hormone concentration in ng/g on the y-axis and sample location from base of baleen plate in cm on the x-axis.

**Fig. 7:**
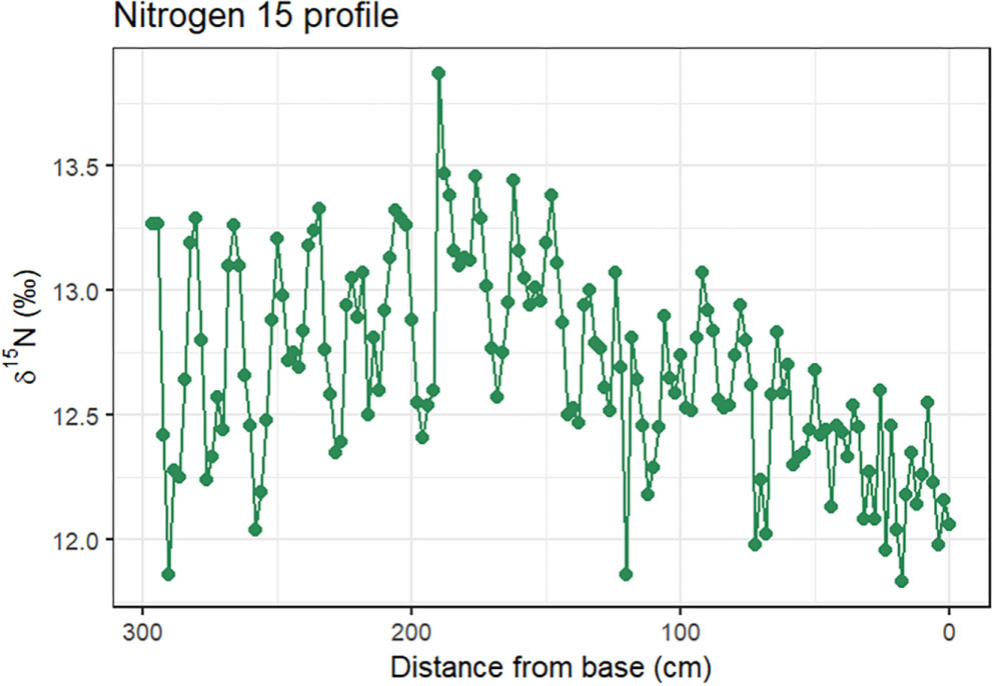
*δ^15^Nitrogen* profile, with stable isotope ratio on the y-axis and sample location from base of baleen plate in cm on the x-axis.

Here we present how to perform a cross-correlation function (CCF) for analysis using the *male bowhead testosterone* (Fig. 6) and *δ^15^Nitrogen* (Fig. 7) datasets. The first step is to detrend both datasets to remove any trend from the time series, we do this using the *diff* function from the ‘stats’ package in R (R Core Team 2024). See Box. 1 for more information on detrending.

**Fig. 8:**
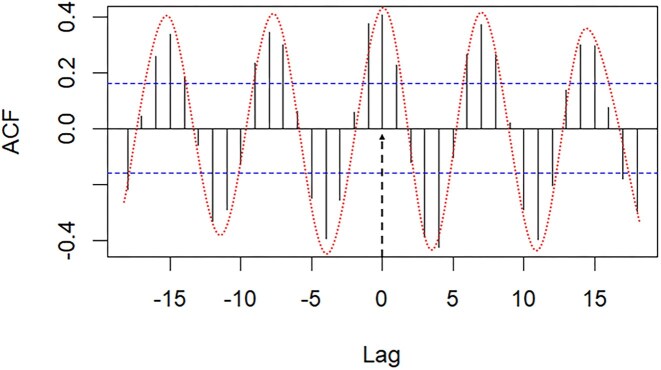
Cross-correlation ACF for testosterone based upon past values of *δ^15^N*. The blue-dotted horizontal lines indicate confidence intervals with values above being significant. Red-occilating dotted lines indicate the seasonality in the time series. The arrow shows lag 0 (the same time points for both series).

To fit the cross-correlation function, we use the *ccf* function from the “tseries” package (Trapletti and Hornik 1999) in R. We enter our primary time series as x, in this case testosterone, and our secondary time series (the one potentially influencing x) as y. This will produce an ACF plot (Fig. 8). Lag zero is located in the center of the plot which indicated the concurrent correlation between x and y. Positive lags indicate that *X* leads *Y*, where changes at time *t* in *X* influence *Y* at time *t* + significant lags. Negative lags indicate that *X* lags *Y*, where changes in *X* at time *t* are influenced by previous values of *Y*. On the ACF plot (Fig. 8) for the CCF, spikes that cross the blue-dashed lines indicate autocorrelation values that are statistically significant. Here, we can see that δ^15^Nitrogen and Testosterone are strongly correlated through time, combined with seasonal cycles, indicating both cycle through time with *δ^15^N* following rises in testosterone.

### Spectral analysis

Time series analysis usually considers how the signal in the data changes over time. Spectral analysis instead considers how the signal is distributed within different frequencies, the location of the cycle in time, and bandwidth sizes, the width of the frequency of the cycle (i.e., a frequency domain). Spectral analysis is primarily used in physics and geology; however, it has several uses outside these fields including areas of biology. It looks at the covariance of the time series (i.e., spectral density), which can then be used to estimate the squared correlation between the time series and the waves (a single oscillation) at different frequencies along the time series, which is known as the periodogram (Venables and Ripley 2002). For large time series, the periodogram is relatively independent for distinct frequencies in the data, however, by smoothing the periodogram the high variance in the data can be reduced providing a clearer periodogram.

The periodogram presents peaks at the frequencies of these repeated patterns in the data, representing how often they are repeating. This can provide valuable insights into underlying processes in endocrinology such as the frequency of migration events (as inferred from complementary stable isotope analysis), seasonal breeding patterns from testosterone cycles or repeated frequencies of stress in animals in their cortisol or corticosterone concentrations. More information on the statistical model of the spectral analysis, see the *Supplemental Information—Model details*.

Spectral analysis in practice is much simpler than the theory underpinning it, with modern computational power and statistical software doing most of the calculations behind the scenes. *SI Box 3* provides a worked example of spectral analysis.

### Peak analysis

Peaks are useful topographical features in time series, as they generally have clear, direct interpretations, such as stress with “spikes” in cortisol or corticosterone, pregnancy with rises in progesterone, and even migration with peaks in stable isotope values. As a result, analysis of peaks can provide valuable insight into both regularly recurring events (e.g., migration) and more intermittent ones (e.g., breeding events). Peaks can be broken down into two types: (1) peaks which are narrow, sharp rises followed by a precipitous decline, and (2) elevated values that are broad over time (Palshikar 2009).

One common way to identify peaks is by detecting local maxima by comparing points in the time series to its neighboring points in a set window (i.e., a defined number of time steps) to calculate if the focal point is greater than those surrounding it, i.e. a peak. However, local maxima may not represent true peaks, but noise in the data. Given that noise represents random variation due to natural variability or observation error, we want to exclude it from the interpretation and further analysis of the time series. There are a number of ways to identify true peaks, including calculating a score equivalent to the probability of a point being a true peak, and using a threshold value over which peaks would no longer be considered noise. Both methods require careful thought from the user with regard to how to calculate the score or set the threshold. For example, the *scorepeak* package (Ochi 2019) in R (R Core Team 2024) provides three types of peak function, the choice of which determines how conservative the method is when it comes to the identification of peaks. For the threshold-based method, a commonly used endocrinology package in R, *hormLong* (Fanson and Fanson 2015), uses an iterative process where any points greater than the cutoff value (mean + *z* × standard deviation, where *z* is the number of standard deviations above the mean × SD) are removed, and then the process is repeated until no points exceed the cutoff. At the end of the iterative process, all remaining observations are considered “baseline,” while those that were excluded are defined as peaks. The choice of *z* defines the threshold of these peaks. It should be noted that the value of how many standard deviations on either side of the mean that define the baseline is arbitrary and should be thoroughly considered before implementation to ensure that the baseline accurately represents the “normal” hormone range for each species and demographic group analyzed. However, the choice of *z* could still misclassify low peaks, with some hormones having physiological relevance close to baseline values. The iterative method assumes that individuals spend the majority of time at baseline, which is not always the case (i.e., chronic stress or frequent pregnancies). Worked examples of the peak analyses described can be found in *SI Box 4*.

### Amplitude analysis

The amplitude change in a time series can take two forms, peak-trough amplitude change (the change from the highest value to the lowest value in a cycle), and the classical amplitude change (the change from the mean value to the peaks and troughs of the cycles). These changes can tell us important information about hormone cycling in wildlife populations. For example, increased amplitude of testosterone could indicate commencement of breeding in mature individuals. We will be focusing on peak-trough amplitude change.

The simplest method for measuring a change in amplitude is by identifying the peaks and troughs of the time series (e.g., via peak analysis). Once identified, the distance between each peak and its subsequent trough can be calculated, providing the distance of each amplitude change. A simple chi-squared test can then be done to see if the distances of the amplitude change over the time series. The expected frequency of the chi-squared test would be equal distance between each peak and trough.

Another way to measure changes in amplitude is by looking at the seasonal changes in the dataset, and rather than assuming seasonal changes are constant, considering them as time-varying amplitudes (Holmes et al. 2021). This method assumes the location of the peaks remains constant, however, the difference between the peaks and troughs changes. This model can be fit using a multivariate autoregressive state-space (MARSS) model to calculate the amplitude change. An example of how to fit this model can be found in Box. 4. For more information on this method of amplitude analysis, see the *Supplemental Information—Model details*.

Box 4. Amplitude analysis

Here we present time varying amplitude analysis using the *male bowhead testosterone dataset*. To do this, we use code created by (Holmes et al. 2021). For this analysis, we use the original dataset (no detrending). This method uses a multivariate state-space (MARSS) model.

First, we need to know the frequency of the cyclical pattern in the dataset, if this is not known from the sampling frequency, it can be inferred from additional analyses such as ACF, ARIMA and/or spectral analysis. Using the data and the seasonality information, we can set up the model parameters where we have two curve parameters, one for the sine and the other for the cosine which we will use our number of samples and sampling frequency to estimate, here we had 149 samples and a frequency of 7 estimated from the ACF. Next, we need to set up our Z matrix which will be used to calculate our hidden states (amplitude change) from our data.

Using the data and parameters we defined above, we can fit these to the MARSS model using the *MARSS* function from the ‘MARSS’ package (Holmes et al. 2012) in R (R Core Team 2024) (see *Supplemental Information—code* and *Model details* for in depth details on the model fitting).

**Fig. 9:**
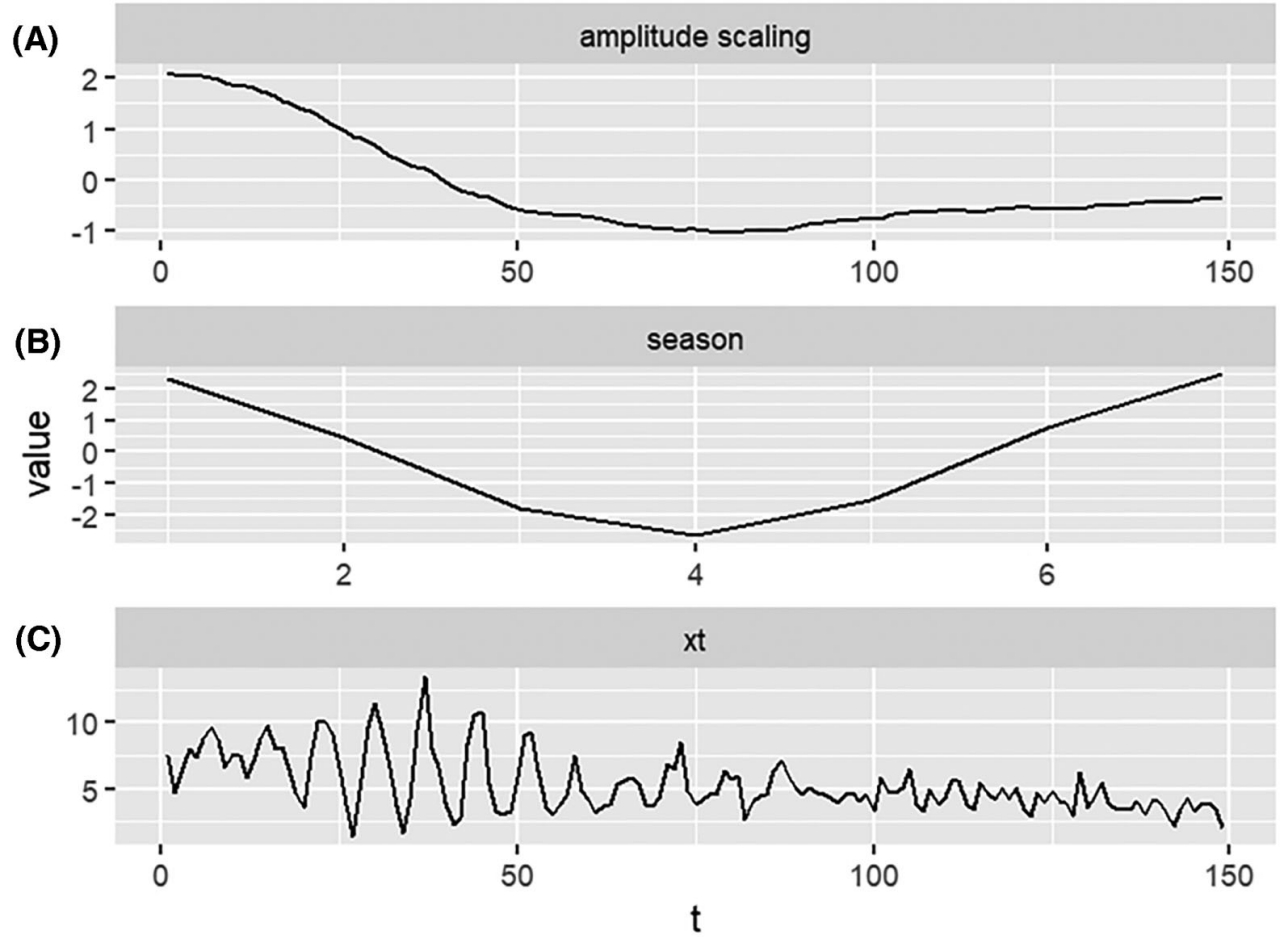
Output from the amplitude analysis model, with the time frame represented on the x-axis. (A) shows the level of amplitude change across the time series, B) shows the seasonal component of the time series, and (C) shows how amplitude changes with relation to the seasonality across the series after its been scaled. Note that t (time) is done by sample number rather than cm, so 1 = 1 cm, 2 = 3 cm, 3 = 5 cm and so on.

Once the model has run, it provides up with several metrics, with the three of interest being the amplitude scaling (the level of amplitude change), season (the seasonal component of amplitude) and *xt* (how the amplitude changes in time with regards to the seasonality) (Fig. 9).

### Additional models

We have presented several different models commonly used in time series analysis. This list, however, is not exhaustive, and there are numerous additional methods available, which we briefly highlight here

#### Change point models

Change point analysis is a useful tool that can identify points in the time series where the properties of the data, such as the mean or variance, change significantly. The key concepts of change point analysis are testing if a change has occurred, locating the point(s) of change, and estimating the degree of the change (Carlstein and Siegmund 1994; Chen et al. 2000; Killick et al. 2010). Change point analysis has been used for detecting climate change, monitoring medical conditions, analyzing human activity, and numerous other applications (Aminikhanghahi and Cook 2017; Van den Burg and Williams 2020). In physiology, change point analysis has been applied to δ^15^N profiles along beluga teeth to detect drops in values indicative of weaning (Matthews and Ferguson 2015b); a similar approach could be used in endocrinological applications such as identifying points along glucocorticoid profiles indicative of chronic or acute stress over time.

#### Autoregressive conditional heteroskedasticity models

Common time series models, such as the ARIMA model assume that the variance is constant over the series; however, this is not always the case, with volatility present in many data streams. Autoregressive conditional heteroskedasticity (ARCH) models allow for the conditional variance to change over time (Engle 1982). The ARCH works by modeling the variance (volatility) of the time series, rather than the mean, as is done in the ARIMA model. This is particularly useful when volatility in the series tends to cluster (periods of high volatility are followed by other periods of high volatility, and the same for low volatility). The ARCH models have typically been used in finance for stock returns and exchange rates; however, they have also been applied to fields such as hydrology (Wang et al. 2005), and ecology (Seekell et al. 2011, 2012) where they have been used to identify regime shifts. In endocrinology, the approach could be useful for modeling changes in foraging ecology from stable isotopes, or for hormones, such as thyroid, which can show large fluctuations due to the myriads of functions in which they are involved (e.g., nutritional stress, thermoregulation).

#### Purpose built models

Previously we discussed standard time series models that can be applied in relatively straightforward ways; however, there are many instances where these methods are not applicable due to the structure of the data, missing values, or specific research questions. It is possible to account for these issues, but it often requires custom methods specific to the issue at hand.

Time series regression models, which explicitly account for autocorrelation in the residuals, as well as being capable of modelling nonlinearity, can be useful when periodicity and frequency vary in the time series data. For example, the Time Warping Model was applied to analyze the stable isotope ratios of narwhal tusks, where both intrinsic and extrinsic factors influence the rate of growth of the tusk at different periods in an individual’s life (Nielsen et al. 2024).

Multivariate analysis is a group of statistical techniques that allows for the simultaneous assessment of multiple variables. The banner of multivariate analysis includes many different statistical methods, such as principal components analysis (PCA), clustering, and multidimensional scaling (MDS). These models make it possible to analyze the relationship between multiple hormone profiles or complementary data such as stable isotopes, movement, or behavior.

### Combining times series models

In the previous sections, we have explained specific single analyses that can be used for time series data, however, to address the specific questions that are being asked as part of physiological or endocrinological studies, a single analysis on its own will not likely be enough. The methods we have presented can be used in combination with each other and additional analyses to answer specific questions. For example, in the study of baleen for stable isotope and hormone analysis (Matthews and Ferguson 2015a; Lysiak et al. 2018; Pomerleau et al. 2018; Hunt et al. 2022) components of the ARIMA model were combined with spectral analysis to understand the frequency of cyclic cycles in the profiles. Using detrended data to remove any linear trends present, the data are then fit to a high-order AR model for each series. The modelled time series are then used to perform spectral analysis to calculate the length of the cyclic cycles. Combining these methods allows for trend and noise to be accounted for, thus better estimating the periodicity of the cycles. This is just one example of how different methods can be combined to address specific questions that may not be possible with the use of a single method on its own.

## Conclusion

As the ability to capture temporal physiological and ecological data from individuals becomes more widely available, the need to analyze time series data becomes increasingly important. By using time series analysis for endocrine data, the structure of how hormones change in individuals and species can be better understood, providing important insights into management, conservation, and physiology.

Here, we have presented some classic time series analysis models and how they can be used with endocrine data to better understand what is happening across the span of the hormone profiles. Many of these models have tools readily available for their application and can help researchers understand the correlation within and between hormone profiles, identify peaks and frequency of peaks, as well as how the amplitude of the data might change over time. We have also presented a number of other useful time series analyses that can be of use for specific questions but may require greater statistical expertise to execute. This is not an exhaustive set of tools, and there are increasingly complex methods for analyzing time series data to address specific questions or issues with data. For individuals new to time series analysis or interested in more of the details behind the methods discussed here, we have provided resources, code, model details and further worked examples in the supplementary information (*Supplemental Information—resources; code; model details*; and *further worked examples*).

## Supplementary Material

icag092_Supplemental_Files

## Data Availability

All data used in the worked examples of this paper are included in the supplemental information.

## Notes

From the symposium “ Hormones at Sea: New Frontiers in Marine Mammal Endocrinology and Ecophysiology” presented at the annual meeting of the Society for Integrative and Comparative Biology, January 3-7, 2026.
