# Supplementary material for: Time Series Analysis for Physiological and Endocrinological Data: A Practical Guide
Source: Integr Comp Biol. 2026 Jun 18;66:icag092. doi: 10.1093/icb/icag092 (PMC13339088; doi:10.1093/icb/icag092)
Supplement: icag092_Supplemental_Files [file icag092_supplemental_files.zip › icb-2026-0050-File011.docx]

Supplemental Information - Model details

# ARIMA models

## **Autoregressive**

In a time series, the partial autocorrelation between an observation *y* at time *t* ($y_{t})$ and previous observations ($y_{t-h})$, where *h* is the number of lags, is the correlation between $y_{t}$ and $y_{t-h}$ conditional on the set of observations between time *t* and *t – h* ( $y_{t-h+1}, \ldots, y_{t-1}$,) (Brandorff-Nielsen & Shou, 1973)*.* The partial autocorrelation for $y_{t-1}$ (i.e. the 1^st^ order partial autocorrelation) is equal to the 1^st^ order autocorrelation (i.e. the correlation between the two points in time). The 2^nd^ order partial correlation is described as

$\frac{Covariance \left( y_{t},y_{t-2} | y_{t-1} \right)}{\sqrt{Variance\left( y_{t-1} \right)Variance\left( y_{t-2} | y_{t-1} \right)}}$,

which is the correlation between the values two time periods apart, conditional on the information provided by the value between. Therefore a 3^rd^ order partial autocorrelation is

$\frac{Covariance \left( y_{t},y_{t-3} | y_{t-1},y_{t-2} \right)}{\sqrt{Variance\left( y_{t-1},y_{t-2} \right)Variance\left( y_{t-3} | y_{t-1},y_{t-2} \right)}}$,

and so forth (ArunKumar and others 2021). These partial autocorrelations are the correlation of the residuals from two different regressions: 1) a regression where we predict $y$ from $y_{t-h}$; and 2) a regression where we predict $y_{t-h}$ from $y_{t-h+1}$, where we correlate the components of $y_{t}$ and $y_{t-h}$ that are not predicted by $y_{t-h+1}$. Thus, the partial autocorrelation function gives the correlation between a specific time point and its previous values at a specific lag, without the influence of the intermediate lags.

This information can be used to fit an autoregressive model (Shibata, 1976). The autoregressive model is expressed as:

$Y_{t}= \beta+ \underline{\phi_{1}y_{t-1}+\phi_{2}y_{t-2}+\ldots+\phi_{p}y_{t-p}}+ \varepsilon_{t}$,

where *Y_t_* is the value of the time series at time *t*, *β* is a constant, $\phi$*_p_* are the autoregressive components of the lagged variables, *y_t_*_-1_, …, *y_t_*_-_*_p_* are the lagged values of the time series, and *ε_t_* is the white noise term (Shaman and Stine 1988; Ullrich 2021).

## **Moving Average**

The autocorrelation function can be expressed as:

$$\hat{p}_{k}= \frac{\sum_{t=k+1}^{T} \left( r_{t}-\underline{r} \right)\left( r_{t-k}-\underline{r} \right)}{\sum_{t=1}^{T} (r_{t}-\underline{r})^{2}}$$

where $\hat{p}_{k}$ is the autocorrelation value for lag *k*, $\underline{r}$ is the mean center of the time series, $r_{t}$is the value of the time series at time *t*, *k* is the lag, and *T* is the length of the time series (Cryer and Chan 2008).

The moving average model is expressed as:

$$Y_{t}= \mu+ w_{t}+\Theta_{1}w_{1-q}+\Theta_{2}w_{2-q}+ \ldots+\Theta_{q}w_{t-q}$$

where *Y_t_* is the value of the time series at time *t*, µ is the mean of the time series, *w_t_* is the error term for the white noise at time *t*, $\Theta$ is the coefficients of the model that determine the weights of each past error term, and *q* is the model order of the moving average (the number of past error terms (Durbin 1959). It should be noted that the model can also be defined with a negative in front of the theta terms, but that there is no difference in the theoretical properties of the model. Its main influence is to flip the algebraic signs of the coefficient values and unsquared theta terms in the formulas for the autocorrelation function and variances.

## **Autoregressive Integrated Moving Average**

If the time series is not stationary, first order differencing can be applied:

$${y^{'}}_{t}= y_{t}- y_{t-1}$$

where *y`_t_* is the value of the differenced time series at time *t*, and *y* is the value of the time series at time *t* (McGonigle and others 2022). If the data are still not stationary, second order differencing can be applied:

$${y^{''}}_{t}= {y^{'}}_{t}- {y^{'}}_{t-1}$$

where *y’’_t_* is the value of the second order differenced time series (McGonigle and others 2022).

The general model structure expressed as:

$$y_{t}\left( d \right) =c+\varepsilon_{t}+\underline{\phi_{1}y_{t-1}^{\left( d \right)}+\phi_{2}y_{t-2}^{\left( d \right)}+\ldots+\phi_{p}y_{t-p}^{\left( d \right)}}+\underline{\theta_{1}w_{t-1}+\theta_{2}w_{t-2}+\ldots+\theta_{q}w_{t-q}}$$

where *d* represents the integrated component (the number of times the time series is differenced to make it stationary), *c* is a constant, ε_t_ is the white noise/error term, ϕ are the autoregressive coefficients calculated by the model, *y_t_* is the current value of the time series, *p* is the order of the autoregressive model, *θ* are the moving average coefficients calculated by the model, and *q* is the order of the moving average model (Box and Jenkins 1970; 1976).

# Spectral Analysis

Spectral analysis starts with understanding the spectral density of the time series (*t*). The spectral density is a function that describes the distribution of the variance of a time series across different frequency components. We begin with the equation of the cosine curve:

$$\mu_{t}=A\cos\left( 2\pi wt+ \Phi\right)$$

where:

- *A* (> 0) is the amplitude, as the time (*t*) varies, the curve oscillates between *A* and −*A*.
- *w*, is the frequency, and 1/*w* is the period or cycle. As *t* varies from 0 to 1/*w* the process goes through one full cycle (i.e. it ends where it started).
- *Φ* is the phase of the curve. *Φ* also serves as the arbitrary origin of the time axis.

However, we can introduce randomness into the process by allowing *A* and *Φ* to be random (not all amplitudes and phases need to be equal). To get this, a trigonometric identity can be used to represent the equation as:

$$\mu_{t} =U_{1}\cos\left( 2\pi wt \right)+U_{2}\sin\left( 2\pi wt \right),$$

where

$U_{1}=Acos\left( \Phi\right), U_{2}= -Asin\left( \Phi\right)$,

$$A= \sqrt{U_{1}^{2}+U_{2}^{2}}, \Phi={tan}^{-1}\left( -{U_{2}}/{U_{1}} \right),$$

where for a fixed frequency *w*, *cos(2πwt)* and *sin(2πwt)* are predictor variables and *U_1_* and *U_2_* are two random variables that determine the cosine and sine components separately. The combination of *m* cosine curves can be expressed as (Platt and Denman 1975):

$$Y_{t}= \sum_{j=1}^{m} \left[ U_{j1}\cos\left( 2\pi w_{j}t \right)+U_{j2}\sin\left( 2\pi w_{j}t \right) \right]$$

When the frequencies of interest are of a special form, the regression becomes increasingly easy. If *n* is odd, *n – 2k+1*, where *k* represents the frequency of the sine wave. The frequencies are called Fourier frequencies and take the form 1/n, 2/n, …, k/n. The sine and cosine predictor variables at these frequencies are known to be orthogonal, and the least squares estimates are simply

$$\hat{a}_{j}=\frac{2}{n}\sum_{t=1}^{n} Y_{t}\cos\left( {2\pi j}/{n.t} \right) and \hat{b}_{j}=\frac{2}{n}\sum_{t=1}^{n} Y_{t}\sin\left( {2\pi j}/{n.t} \right)$$

This gives us the exact decomposition

$$x_{t}= \bar{x}+ \sum_{j=1}^{(n-1)/2} (\hat{a_{j}}\cos\left( {2\pi j}/n.t \right)+\hat{b_{j}}\sin\left( {2\pi j}/n.t \right)), t=1,\ldots,n$$

Using these equations, we can now calculate the periodogram (*P_x_*), which is a visual tool used for identifying the dominant frequencies and periodic patterns in time series data (Schuster 1897). The periodogram takes the frequency *w = j/n* for *j = 1, 2, …, k* is defined as

$$P_{x}\left( j/n \right)=\frac{n}{4}\left( \hat{a}_{j}^{2}+\hat{a}_{j}^{2} \right)$$

Large values of the periodogram represent the predominant frequencies in the series.

# Amplitude analysis

Seasonal cycles in a time series aren’t always constant, and the amplitude of these peaks can change while the location remains the same. Similar to the spectral analysis, time-varying amplitude (Holmes and others 2021) can be expressed as

$z_{t}\left( \beta_{1}\sin\left( \frac{2\pi t}{p} \right)+\beta_{2}\cos\left( \frac{2\pi t}{p} \right) \right)$,

where *z_t_* is the sinusoidal curve of the time series at time *t*, *β* determines the shape and amplitude of the seasonal cycles, and p is the frequency of the data. For this model, the values of *β* remain constant, while the sum of the sine and cosine components are multiplied by a time varying scaling factor. This model for seasonality, however, is underdetermined (meaning there are too many unknowns in the equation) due to the need to calculate the two *β* values and *z_t_*. This can be amended by dividing *z_t_* and *β_2_* by *β_1_* and multiplying this by the seasonal component of the model. The seasonal model can then be expressed as

$${(z}_{t}/\beta_{1})(sin\left( \frac{2\pi t}{p} \right) +{(\beta}_{2}/\beta_{1})(cos\left( \frac{2\pi t}{p} \right) )=$$

$$x_{2,t}\left( \sin\left( \frac{2\pi t}{p} \right)+\beta\cos\left( \frac{2\pi t}{p} \right) \right)$$

The seasonal peaks between the two equations are the same, the difference is in the amplitude, which is determined using *x_2, t_*.

*x_2, t_* is a hidden state and can be inferred using a *Z* matrix which links the observations to these hidden states. The *Z* matrix for this model is

$$Z = \left[ \begin{matrix} x_{1} \\ x_{2} \end{matrix} \right]_{t}=\left[ \begin{matrix} x_{1} \\ x_{2} \end{matrix} \right]_{t-1}+\left[ \begin{matrix} w_{1} \\ w_{2} \end{matrix} \right]_{t}$$

where *x* is the hidden states and *w* are discrete white noise series. The model for the data (*y_t_*) can then be expressed as

$$y_{t}= \left[ 1\sin\left( \frac{2\pi t}{p} \right)+\beta\cos\left( \frac{2\pi t}{p} \right) \right] \left[ \begin{matrix} x_{1} \\ x_{2} \end{matrix} \right]_{t} + v_{t}$$

$$v_{t}\sim Normal\left( 0,\eta^{2} \right)$$

Where *v_t_* is a bias parameter.

# References

ArunKumar, K.E., Kalaga, D.V., Kumar, C.M.S., Chilkoor, G., Kawaji, M., and Brenza, T.M. (2021). Forecasting the dynamics of cumulative COVID-19 cases (confirmed, recovered and deaths) for top-16 countries using statistical machine learning models: auto-regressive integrated moving average (ARIMA) and seasonal auto-regressive integrated moving average (SARIMA). *Applied Soft Computing*. **103**: 107161. <https://doi.org/10.1016/j.asoc.2021.107161>.

Box GEP, Jenkins GM (1970) Time series analysis, forecasting, and control. Holden-Day, Oakland

Box, G., & Jenkins, G. M. (1976). Analysis: Forecasting and control. San Francisco, 10.

Brandorff-Nielsen, O., and Schou, G. (1973). On the parametrization of autoregressive models by partial autocorrelation. *Journal of Multivariate Analysis*. **3**: 408-419. <https://doi.org/10.1016/0047-259X(73)90030-4>.

Cryer, J.D., and Chan, K. (2008). Chapter 6: model specification. In G. Casella, S. Fienberg, & I. Okin (Eds.), *Time Series Analysis: With Applications in R (2nd Edition)* (109-148). Springer. doi:[10.1007/978-0-387-75959-3](https://link.springer.com/book/10.1007/978-0-387-75959-3).

Durbin, J. (1959). Efficient Estimation of parameters in moving-average models. *Biometrika*. **46**(3/4): 306-316. <https://doi.org/10.2307/2333528>.

Holmes, E.E., Sheuerell, M.D., and Ward, E.J. (2021). Time-varying amplitude. In *Applied time series analysis for fisheries and environmental data*. Edition 2021.

Platt, T., and Denman, K.L. (1975). Spectral analysis in ecology. *Annual Review of Ecology and Systematics*. **6**: 189-210. https://www.jstor.org/stable/2096830.

McGonigle, E.T., Killick, R., and Nunes, M.A. (2022). Modelling time-varying first and second-order structure of time series via wavelets and differencing. *Electronic Journal of Statistics*. **16**: 4398-4448. https://doi.org/10.1214/22-EJS2044.

Schuster, A. 1897. On lunar and solar periodicities of earthquakes. *Proc. R. Soc.* 61 (369-377): 455–465. <https://doi.org/10.1098/rspl.1897.0060>

Shaman, P., and Stine, R.A. (1987). The bias of autoregressive coefficient estimators. *Journal of the American Statistical Association*. **83**(403): 842-848. https://doi.org/10.1080/01621459.1988.10478672

Shibata, R. (1976). Selection of the order of an autoregressive model by Akaike’s information criterion. *Biometrika.* **63**(1): 117-126. <https://doi.org/10.1093/biomet/63.1.117>.

Ullrich, T. (2021). On the autoregressive time series model using real and complex analysis. *Forecasting*. **3**(4): 716-728. <https://doi.org/10.3390/forecast3040044>.
