# Supplementary material for: Time Series Analysis for Physiological and Endocrinological Data: A Practical Guide
Source: Integr Comp Biol. 2026 Jun 18;66:icag092. doi: 10.1093/icb/icag092 (PMC13339088; doi:10.1093/icb/icag092)
Supplement: icag092_Supplemental_Files [file icag092_supplemental_files.zip › icb-2026-0050-File012.pdf]

Supplemental Information - Further worked examples

SI Box 1. Moving Average

Here we present how to conduct a moving average model using the *male bowhead testosterone* data (SI Fig. 1). The first step is to detrend to remove the trend from the data, we did this through differencing using the *diff* function from the \`stats\` function in R (R Core Team 2024). We set the number of differences as 1. If you are interested in removing the seasonal component, you can also seasonally difference in this function using the \`lag\` argument.
Once the dataset is stationary, we can fit the ACF using the *acf* function from the \`tseries\` package (Trapletti and Hornik 2026) in R (R Core Team 2024). The ACF will produce a plot (SI Fig. 2) where spikes that cross the blue dashed lines indicate autocorrelation values that are statistically significant. The highest lag with a significant spike can be used as your model order in the autoregressive model.
Using the model order, we can now fit the moving average model using the *arima* function from the \`stats\` package in R (R Core Team 2024), where we set q in the \`order\` equal to our model order (in this case 21). The *arima* function has three arguments for \`order\`, (p – autoregressive lag order, d – integrated component for the amount of differencing, and q – moving average lag order), where we only need to set the value for q for the moving average model and the other two as zero. The output of the autoregressive model can then be used to predict future values using the *forecast* function from the \`forecast\` (Hyndman and Khandakar 2008) in R or used in conjunction with other analyses to address specific questions with the predicted data.

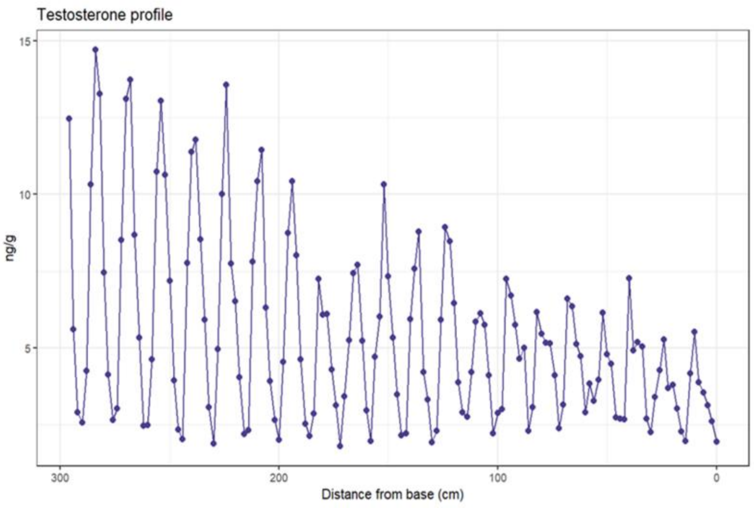

SI Figure 1. Testosterone profile for male bowhead whale with concentration in ng/g on the y-axis, and sample location as distance from the base in cm on the x-axis

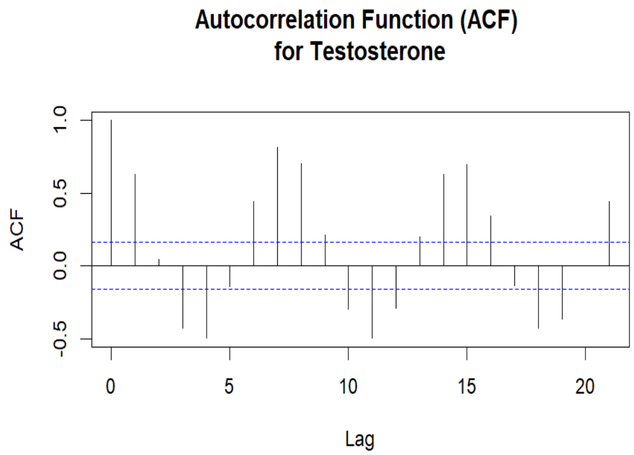

SI Figure 2. The autocorrelation function for the testosterone profile, with lags on the x-axis and correlation on the y-axis. The horizontal dotted lines indicate the confidence intervals, with values over the line indicating significant lags

SI Box 2. ARIMA model

Here we present how to perform an ARIMA model using the *male bowhead testosterone* data. For the ARIMA model, the data does not have to be detrended due to the Integrated (I) component which can do the differencing in the model. This means that we can either manually detrend our data or include the amount of differencing we wish to include in the model.

Like the AR and MA models, we need to conduct both the PACF and ACF functions to see the number of lags (model order) to include in the AR and MA components of the model, if they are needed. The ARIMA model does not need to contain values for all the components if they are not needed and can be substituted with zeros if not needed. Looking at the PACF (SI Fig. 3A) we need three AR lags, and two from the ACF (SI Fig. 3B) for the MA component. For the PACF and ACF, spikes that cross the blue dashed lines indicates correlation values that are statistically significant.

Once we have identified all the components, we can fit the ARIMA model using the *arima* function from the \`stats\` package in R (R Core Team, 2024). We set our time series data as the first argument and then for the "order" argument we need to concatenate three numbers (c(AR,I,MA)) where the first is the number of lags from the AR component, the second is the amount of differences, and the third is the number of lags for the MA component. If there is seasonality in the model, you can use the "seasonal" and "period" arguments to specify the model. The output of the ARIMA model can then be used in conjunction with other analyses to address specific questions, or to predict future values from the series using the *forecast* function from the \`forecast\` (Hyndman and Khandakar 2008) package in R.

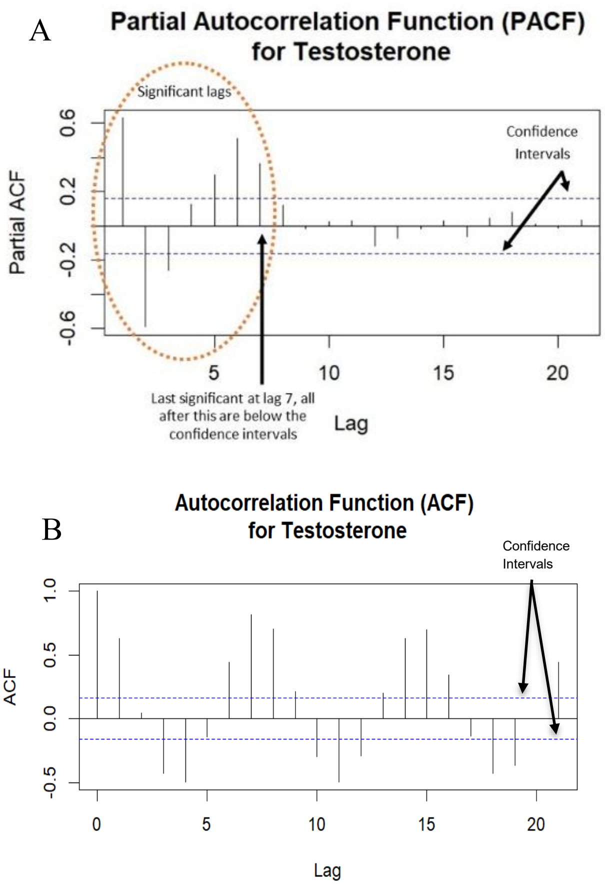

SI Figure 3. The A) partial autocorrelation function and B) autocorrelation function for the testosterone profile, with lags on the x-axis and correlation on the y-axis. The horizontal dotted lines indicate the confidence intervals, with values over the line indicating significant lags

SI Box 3. Spectral Analysis

In this section we will explain how to perform spectral analysis using the *male bowhead testosterone* data. We start off with detrended data to remove the trend from the data and to make it stationary for the analysis. Now we can conduct the spectral analysis on the detrended data using the `spectrum` function from the stats package in R (R Core Team, 2024). The results from this will provide us with our frequencies (how often in the time series the spectral patterns are repeating) and the spectral densities (the amount that each frequency repeats during the time series). Frequencies with high spectral densities mean that those distances between amplitudes are the most common in the time series.

To produce the periodogram, we can take the output of the model for the frequency and spectral density. For the frequency, we need to divide it by the sampling frequency (how frequent the observations where is relation to each other in the data used in the analysis). For the spectral density, we multiply it by two so that the area under the periodogram equals the variance of the time series. We can now use this information to plot the periodogram (SI Fig 4), which shows us that the frequency with the highest spectral density is 0.14. To find out the frequency from our data, we:

$$(1/0.14)x2$$

where we divide the frequency by one and then multiply it by the frequency of the sampling, in this case two, as samples were collected every 2 cm along the baleen plate. This gives us a frequency of cyclical cycles of ~ 14.286 cm for the testosterone data.

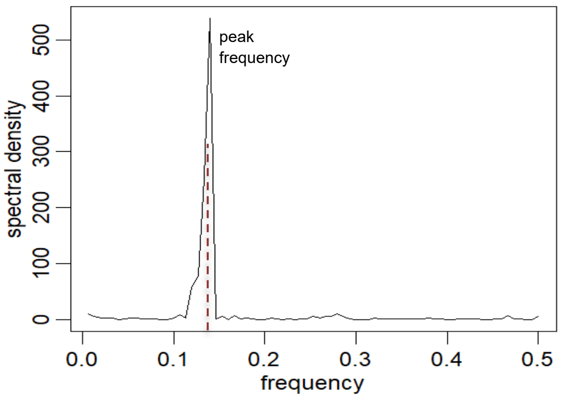

SI Figure 4. The periodogram for the testosterone data showing the peak frequency at 0.14 (dotted line), equal to ~14.286 cm between cyclical peaks in the testosterone data.

SI Box 4. Peak Analysis

In this section we will cover two methods for detecting peaks using the *male bowhead testosterone* data.

hormLong

The first method is the package `hormLong` by Fanson and Fanson (2015) in R (R Core Team 2024). In this method we import our data ensuring that sampling time/frequency, individual ID, hormone and concentrations are all in separate columns. We then use the hormBaseline function to calculate the baseline value for our sample, this is how many standard deviations away from the mean we expect normal concentrations for that hormone to lie within. For the testosterone data we set it at 2SD. From here we can then use the hormArea function which will create a figure (SI Fig 5) where the identified peaks above baseline are numerically identified and greyed out. More details and the code on how to conduct this can be found in *Supplemental Information - code* and at: https://github.com/bfanson/hormLong/blob/master/instructions/hormLong%20instruction%20manual%20v2.pdf.

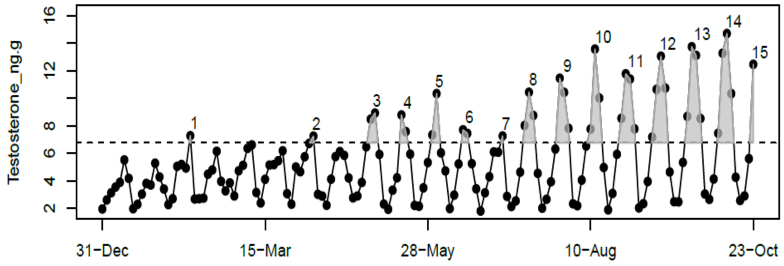

SI Figure 5. Peaks identified using hormLong. Peaks are shown with numbers and are greyed out.

Scorepeak

The second method uses the `scorepeak` package by Ochi (2019) in R (R Core Team 2024). In this method we use the detect_localmaxima function to identify peaks in the data within a set window on either side of each data point, for the testosterone data we set the window to five (this looks at the two points before and after each datapoint). We can then use the score_type1 function to score the peaks to determine if they are peaks or noise. Using these two functions we can identify those locations that were selected by both methods and identify them as peaks.
We can then plot this using the `ggplot2` package (Wickham 2016) in R (R Core Team 2024), where we highlight the peaks identified with red vertical lines (SI Fig 6). More details and the code on how to conduct this can be found in *Supplemental Information - code*.

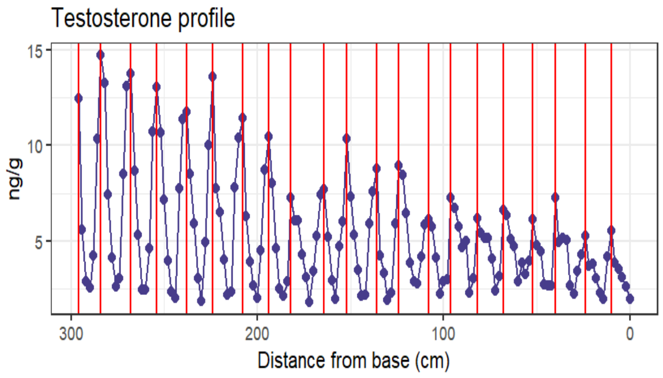

SI Figure 6. Peaks identified using scorepeak. The location of each identified peak is represented by the red vertical line going through the point.

References

Fanson. B., and Fanson, K.V. (2015) *hormLong*: An *R* package for longitudinal data analysis in wildlife endocrinology studies. *PeerJ* PrePrints 3:e1926 https://dx.doi.org/10.7287/peerj.preprints.1546v1

Hyndman, R.J., and Khandakar, Y. (2008). Automatic time series forecasting: the forecast package for R. *Journal of Statistical Software*, **27**(3), 1–22. doi:10.18637/jss.v027.i03.

Ochi, S. (2019). Scorepeak: Peak functions for peak detection in univariate time series. R package version 0.1.2, doi:10.32614/CRAN.package.scorepeak.

R Core Team. (2024). R: A Language and Environment for Statistical Computing. R Foundation for Statistical Computing, Vienna, Austria. https://www.R-project.org/.

Trapletti, A., and Hornik, K. (2026). *tseries: Time Series Analysis and Computational Finance*. R package version 0.10-59, https://CRAN.R-project.org/package=tseries.

Wickham, H. (2016). *ggplot2: Elegant Graphics for Data Analysis*. Springer-Verlag New York. ISBN 978-3-319-24277-4, https://ggplot2.tidyverse.org.
