# Supplementary material for: Time Series Analysis for Physiological and Endocrinological Data: A Practical Guide
Source: Integr Comp Biol. 2026 Jun 18;66:icag092. doi: 10.1093/icb/icag092 (PMC13339088; doi:10.1093/icb/icag092)
Supplement: icag092_Supplemental_Files [file icag092_supplemental_files.zip › icb-2026-0050-File013.docx]

Supplemental Information - code

##### Supplemental Information - code for

###### Time series analysis: a practical guide for endocrinology

This supplemental material will go through the code that has been described in the classical time series analysis section of the manuscript including the boxes. we have divided the code into the same headings used in the manuscript.

# Data

The data that will be used in this code as examples of how to perform the different analyses will primarily be the male bowhead testosterone data:

male.bowhead.testosterone <- read.csv("C:/**FILE LOCATION**/male bowhead testosterone.csv")

# and an additional data set will be used for some of the analyses, the `male bowhead δ15Nitrogen` data:
male.bowhead.δ15Nitrogen <- read.csv("C:/**FILE LOCATION**/male bowhead δ15Nitrogen.csv")

for simplicity sake, we have divided the two separate time series into individual data sets, however, they can be in the same data set and just extracted out as needed. It is important to note, that most time series analyses are conducted on individuals for a specific variable, so if you have multiple individuals sampled, the same analysis will need to be conducted for each separately.

For the bowhead data, due to the way they are sampled, the first sample actually from the end of the individuals life, so to get the data in chronological order, we just need to reverse the time series using the rev function.

reversed_ts <- rev(male.bowhead.testosterone$Testosterone_ng.g)

# Section.1 Time series analysis

### Trend - Seasonality - Remainder

Firstly we make the testosterone data into a time series object using the ts function. We enter the data as the first argument, start is the time of the first sample (this could be day, month, year, or position such as the baleen samples). If multiple samples come from the same period, say year, but different months, you can set start as c(1,2), where “1” is the first period of the samples, say year, and “2” is the time in the period that the sample is from, say 3 if the sample was from March. If the sampling frequency is known, say a sample collected every month across multiple years, you can set the frequency argument to be 12 for the number of months in a year, and so on depending on for different sampling frequencies. NOTE - If the sampling frequency is unknown, it can be obtained visually, or by using an ACF or periodogram (see below for more details on there methods). If there is no detectable frequency in the data set, the frequency does not need to be included.

acf(reversed_ts) # check for the frequency of significant cycles.


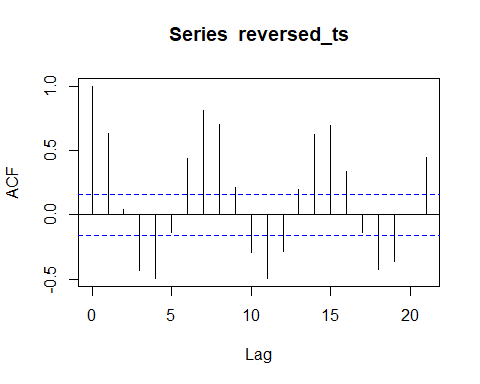


# For the testosterone data, we set the start period as 1, being the first centimeter,
# and frequency as 7, with roughly 14 centimeter seasonal cycles.
ts_data <- ts(reversed_ts, start = 1, frequency = 7)

ts_data # this prints out the time series to make sure everything looks normal.

## Time Series:
## Start = c(1, 1)
## End = c(22, 2)
## Frequency = 7
## [1] 12.46 5.60 2.89 2.56 4.25 10.33 14.70 13.27 7.45 4.12 2.64 3.03
## [13] 8.52 13.11 13.74 8.67 5.33 2.46 2.47 4.63 10.73 13.05 10.64 7.17
## [25] 3.94 2.33 2.02 7.77 11.39 11.77 8.53 5.92 3.07 1.88 4.96 10.01
## [37] 13.57 7.75 6.52 4.04 2.18 2.32 7.81 10.42 11.45 6.30 3.92 2.65
## [49] 2.00 4.53 8.74 10.43 8.01 4.62 2.53 2.12 2.86 7.25 6.07 6.09
## [61] 4.29 3.12 1.79 3.41 5.24 7.44 7.70 5.22 2.95 1.97 4.71 6.02
## [73] 10.33 7.33 5.32 3.48 2.14 2.20 5.93 7.57 8.78 4.21 3.31 1.92
## [85] 2.30 5.91 8.92 8.47 6.46 3.87 2.89 2.75 4.20 5.84 6.12 5.75
## [97] 4.11 2.21 2.88 3.01 7.24 6.71 5.74 4.65 5.00 2.29 3.06 6.17
## [109] 5.45 5.17 5.14 4.10 2.38 3.14 6.60 6.35 5.12 4.72 2.89 3.84
## [121] 3.26 3.95 6.14 4.78 4.47 2.73 2.69 2.67 7.27 4.92 5.18 5.04
## [133] 2.69 2.26 3.40 4.27 5.27 3.69 3.80 3.02 2.28 1.96 4.17 5.51
## [145] 3.88 3.54 3.12 2.60 1.94

Next we can plot the time series data to see if there is any trend or seasonality. using the stl function that is available in the stats package. NOTE - the stl function only works for data with set frequencies, if your data does not have known frequencies, you can use linear regression (explained below), to see if there is a trend in the data.

timeseriescomponents <- stl(ts_data, s.window = 7) # s.window is the sampling frequency
# next we can plot the `stl` to see the components
plot(timeseriescomponents)


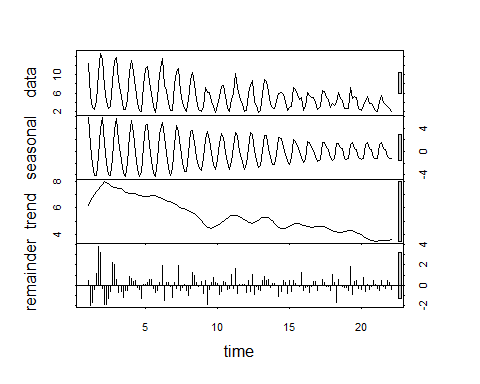


# for non-seasonal data, we plot a linear regression and see if there is a significant
# trend. To do this we will need the "forecast" package.
library(forecast) # load the forecast package - use: install.package("forecast")

## Registered S3 method overwritten by 'quantmod':
## method from
## as.zoo.data.frame zoo

ts_lm <- tslm(ts_data ~ trend) # using the `tslm` package to fit the model looking for
 # trend in the time series.
summary(ts_lm) # the summary of the time series will tell us is there is a significant

##
## Call:
## tslm(formula = ts_data ~ trend)
##
## Residuals:
## Min 1Q Median 3Q Max
## -4.8101 -1.8604 -0.0486 1.5393 7.4145
##
## Coefficients:
## Estimate Std. Error t value Pr(>|t|)
## (Intercept) 7.48282 0.45475 16.455 < 2e-16 ***
## trend -0.02819 0.00526 -5.359 3.16e-07 ***
## ---
## Signif. codes: 0 '***' 0.001 '**' 0.01 '*' 0.05 '.' 0.1 ' ' 1
##
## Residual standard error: 2.762 on 147 degrees of freedom
## Multiple R-squared: 0.1635, Adjusted R-squared: 0.1578
## F-statistic: 28.72 on 1 and 147 DF, p-value: 3.16e-07

# trend in the data.

## Differencing

Once we have visually inspected for a trend, we can formally test this using the Augmented Dickey-Fuller (ADF) and Kwiatkowski–Phillips–Schmidt–Shin (KPSS) tests which test if the time series is stationary, i.e. no trend. These tests are available in the tseries package.

library(tseries)
# Use ADF test (Null hypothesis: non-stationary)
adf.test(ts_data)

## Warning in adf.test(ts_data): p-value smaller than printed p-value

##
## Augmented Dickey-Fuller Test
##
## data: ts_data
## Dickey-Fuller = -4.9479, Lag order = 5, p-value = 0.01
## alternative hypothesis: stationary

# Use KPSS test (Null hypothesis: stationary)
kpss.test(ts_data)

## Warning in kpss.test(ts_data): p-value smaller than printed p-value

##
## KPSS Test for Level Stationarity
##
## data: ts_data
## KPSS Level = 1.5956, Truncation lag parameter = 4, p-value = 0.01

# If your data is non-stationary, but doesn't have a linear trend, but rather has
# a non-zero mean, you can use the `urca` package where you can fit a ADF test,
# but have more control of the type of stationarity using the ur.df function.
library(urca)

## Warning: package 'urca' was built under R version 4.4.2

test <- ur.df(ts_data, type = "drift") # type is equal to "drift" for a non-zero mean
summary(test)

##
## ###############################################
## # Augmented Dickey-Fuller Test Unit Root Test #
## ###############################################
##
## Test regression drift
##
##
## Call:
## lm(formula = z.diff ~ z.lag.1 + 1 + z.diff.lag)
##
## Residuals:
## Min 1Q Median 3Q Max
## -4.2109 -1.0150 -0.1786 1.0251 4.3730
##
## Coefficients:
## Estimate Std. Error t value Pr(>|t|)
## (Intercept) 3.01993 0.31485 9.592 <2e-16 ***
## z.lag.1 -0.56182 0.05236 -10.730 <2e-16 ***
## z.diff.lag 0.63595 0.06140 10.358 <2e-16 ***
## ---
## Signif. codes: 0 '***' 0.001 '**' 0.01 '*' 0.05 '.' 0.1 ' ' 1
##
## Residual standard error: 1.71 on 144 degrees of freedom
## Multiple R-squared: 0.5234, Adjusted R-squared: 0.5168
## F-statistic: 79.08 on 2 and 144 DF, p-value: < 2.2e-16
##
##
## Value of test-statistic is: -10.7299 57.565
##
## Critical values for test statistics:
## 1pct 5pct 10pct
## tau2 -3.46 -2.88 -2.57
## phi1 6.52 4.63 3.81

# In the summary we need to look at the significant levels at the bottom of the output
# to check significance from the critical values.
# To difference the data - make it stationary - we can use the `diff` function.
diff_data <- diff(ts_data)
plot(diff_data) # plot the differenced data to see if it is stationary


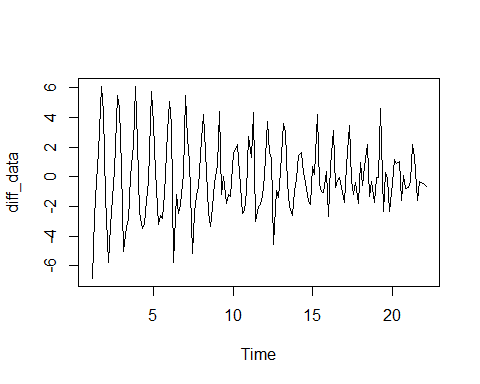


## Seasonal differencing

To remove the seasonal component from the time series, we also use thediff function but we set lag to be the seasonal frequency.

ts_seasdiff <- diff(ts_data, lag=frequency(ts_data), differences=1) # seasonal differencing
plot(ts_seasdiff)


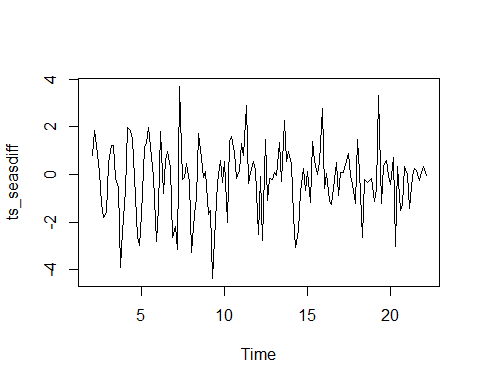


# To plot both the differenced and seasonally differenced data together
par(mfrow = c(2, 1)) # set par to the number of rows first and columns second
plot(diff_data)
plot(ts_seasdiff) # plot your objects


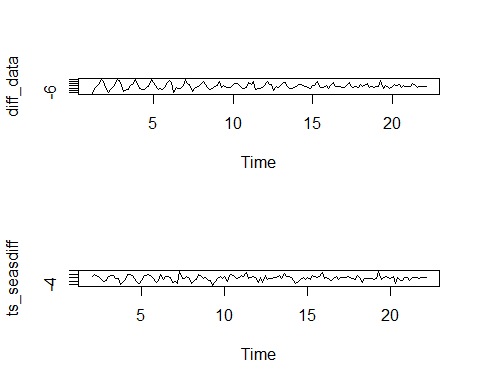


par(mfrow = c(1, 1)) # reset par to normal

# Both seasonal and trend differencing can be done to the times series to make it
# stationary. Another way if you want to remove both of these components is to use
# the remainder object that was created using the `stl` function.
remainder_component <- timeseriescomponents$time.series[, "remainder"]

# Section.2 ARIMA

In this section we will cover three models, the autoregressive (AR), moving average (MA) and the autoregressive integrated moving avereage (ARIMA) which uses the first two (AR and MA) models. We will be using the male bowhead testosterone data for all three examples. ## Autoregressive models ####

### PACF

The first step of the autoregressive model is to conduct the pacf to determine the level of autocorrelation and define the model order needed to fit the model.

pacf(ts_data,
 main = "Partial Autocorrelation Function (PACF) \nfor Testosterone", # Change the main title
) # For this we use the detrended data if it was not stationary


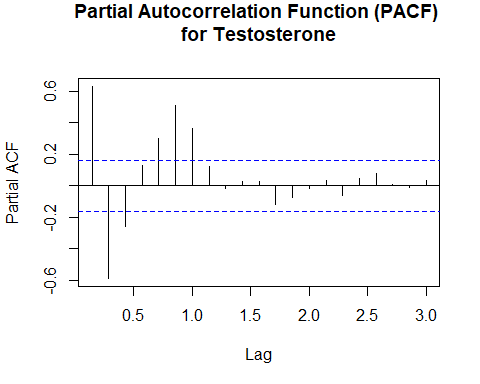


# Fit an AR model with automatic order selection
# The chosen order will be available in fit$order
fit_auto <- ar(ts_data, method = "yule-walker")
# View the results and selected order
print(fit_auto)

##
## Call:
## ar(x = ts_data, method = "yule-walker")
##
## Coefficients:
## 1 2 3 4 5 6 7 8
## 0.4614 -0.0933 -0.1102 0.0061 -0.0781 0.2647 0.3037 0.1214
##
## Order selected 8 sigma^2 estimated as 2.003

fit_auto$order

## [1] 8

## Moving Average models

## ACF

acf(ts_data,
 main = "Autocorrelation Function (ACF) \nfor Testosterone", # Change the main title
)


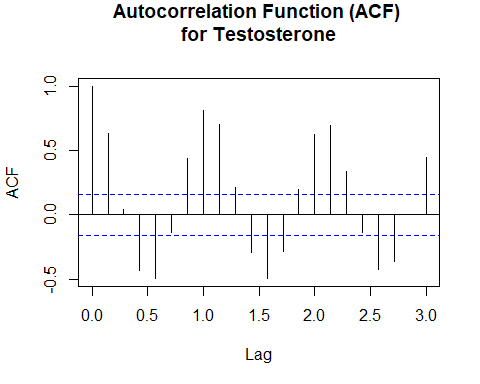


# Fit an MA(1) model to the data
# order = c(p, d, q) where p=AR order, d=differencing order, q=MA order
fitted_model <- arima(ts_data, order = c(0, 0, 21))
fitted_model # view the results of the MA model

##
## Call:
## arima(x = ts_data, order = c(0, 0, 21))
##
## Coefficients:
## ma1 ma2 ma3 ma4 ma5 ma6 ma7 ma8 ma9
## 0.4807 0.0812 -0.3694 -0.2386 0.0298 0.4894 0.6559 0.6768 0.0383
## s.e. 0.0931 0.1264 0.1169 0.1106 0.1457 0.1050 0.1305 0.1182 0.1350
## ma10 ma11 ma12 ma13 ma14 ma15 ma16 ma17
## -0.4146 -0.2835 0.1338 0.3793 0.6729 0.7267 0.1453 -0.3792
## s.e. 0.1173 0.1136 0.1341 0.1550 0.1012 0.1460 0.1346 0.1083
## ma18 ma19 ma20 ma21 intercept
## -0.1962 0.3386 0.3086 0.2871 5.3867
## s.e. 0.1065 0.1150 0.1163 0.0872 0.3894
##
## sigma^2 estimated as 1.213: log likelihood = -237.54, aic = 521.07

## ARIMA models

Here we will cover the ARIMA model which will combine the autoregressive and moving average components covered above, while introducing an integrated component (I). The integrated component can deal with stationarity in the data, by differencing. If the data is already stationary, then the model is an ARMA, the same model, just excluding the need for differencing. The simplest way to fit an ARIMA model is through the forecast package, using the auto.arima function. This function runs multiple ARIMA models to look for the number of autoregressive and moving average lags to include in the model and if differencing is needed. The function compares the different models using AIC and then selects the best fit.

library(forecast) # load the package
testo_arima <- auto.arima(ts_data) # run the auto.arima on the raw data

we can see from this that there is drift in the data, meaning it is not stationary so the model did the differencing and then selected three autoregressive and two moving average lags to include in the model. We can also manually run an ARIMA similar to what we did in the MA section Firstly we need to do our ACF and PACF to see what the model order is, from previously we can use 7 for the AR and 21 for the MA components and because the data has already been differenced and is stationary, we can set the integrative part to 0. When fitting the ARIMA model the order is the same as the acronym AR-I-MA

arima_model <- arima(ts_data, order = c(7, 0, 21)) # run the arima model

## Warning in arima(ts_data, order = c(7, 0, 21)): possible convergence problem:
## optim gave code = 1

arima_model # view the results of the model

##
## Call:
## arima(x = ts_data, order = c(7, 0, 21))
##
## Coefficients:
## ar1 ar2 ar3 ar4 ar5 ar6 ar7 ma1
## -0.0330 0.5735 -0.4643 0.0714 -0.0428 0.1132 0.7292 0.4495
## s.e. 0.1766 0.1728 0.1944 0.3064 0.1591 0.1966 0.1376 0.2106
## ma2 ma3 ma4 ma5 ma6 ma7 ma8 ma9
## -0.4142 0.2729 0.0088 -0.0233 0.0328 -0.2902 0.3374 0.0205
## s.e. 0.1710 0.2391 0.2589 0.1507 0.2135 0.1388 0.1092 0.1264
## ma10 ma11 ma12 ma13 ma14 ma15 ma16 ma17 ma18
## -0.2335 0.0570 0.0888 -0.1281 0.0871 0.3628 0.0429 -0.1520 0.1208
## s.e. 0.1260 0.1314 0.1498 0.1384 0.1565 0.1181 0.1831 0.1543 0.1552
## ma19 ma20 ma21 intercept
## 0.1592 -0.0753 -0.1244 5.5865
## s.e. 0.1837 0.1362 0.1639 1.4074
##
## sigma^2 estimated as 0.7866: log likelihood = -204.23, aic = 468.47

# Section.3 Cross correlation

we start by getting our nitrogen data and testosterone data that we will be comparing in this example. We need to reverse the nitrogen data, and for simplicity we will give them similar names so we an keep track.

reversed_test_ts <- rev(male.bowhead.testosterone$Testosterone_ng.g) # reverse the data
ts_test_data <- ts(reversed_test_ts, start = 1) # make the testosterone data a time series
reversed_nit_ts <- rev(male.bowhead.δ15Nitrogen$d15N) # reverse the data
ts_nit_data <- ts(reversed_nit_ts, start = 1) # make the nitrogen data a time series

# Firstly we need to see if there is a trend in the secondary time series, and make
# that stationary if it is not following the steps in section 1.
Test_diff_data <- diff(ts_test_data) # we can difference the nitrogen data
plot(Test_diff_data) # plot to see if it is stationary


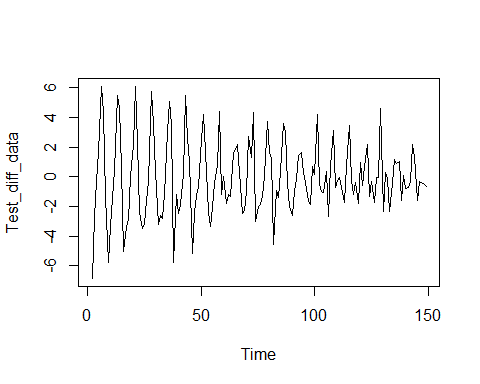


Nit_diff_data <- diff(ts_nit_data) # we can difference the nitrogen data
plot(Nit_diff_data) # plot to see if it is stationary


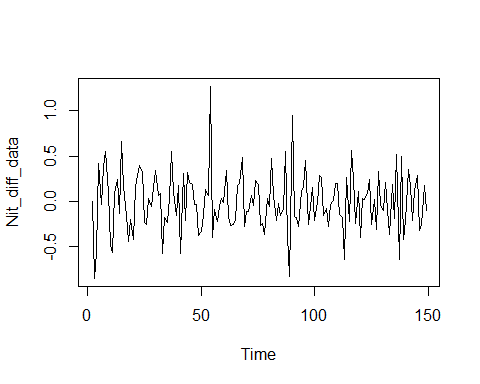


# now we can use this data to conduct our ccf using the `ccf` function
# the first object is our primary data set, followed by the one we are testing
# to see if it can help predict the first. na.action just lets to function skip
# NA's if they are present in the data, and main sets the title.
ccf(Test_diff_data, Nit_diff_data, na.action = na.pass, main = "Cross-correlation Function (CCF) for\n Testosterone and Nitrogen 15") # na.pass handles potential NAs from filtering


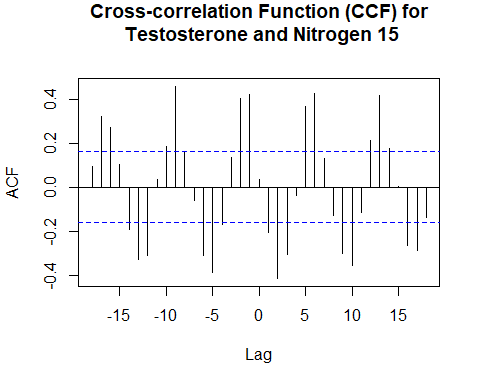


# Section.4 Peak analysis

## hormLong

hormLong is a pakage created by Fanson & Fanson (2015) which we will use for this section on peak analysis. For a full description of the package and all its functions please visit: <https://github.com/bfanson/hormLong/blob/master/instructions/hormLong%20instruction%20manual%20v2.pdf>

library(hormLong) # load the hormLong package
# for this we will use the full data set, which contains:
# - individual identification
# - time of sample - in our case distance from base of baleen the sample was taken
# - hormone concentrations
male.bowhead.testosterone # the data set we are using

## Individual Dist_from_base_cm Testosterone_ng.g
## 1 8 0 1.94
## 2 8 2 2.60
## 3 8 4 3.12
## 4 8 6 3.54
## 5 8 8 3.88
## 6 8 10 5.51
## 7 8 12 4.17
## 8 8 14 1.96
## 9 8 16 2.28
## 10 8 18 3.02
## 11 8 20 3.80
## 12 8 22 3.69
## 13 8 24 5.27
## 14 8 26 4.27
## 15 8 28 3.40
## 16 8 30 2.26
## 17 8 32 2.69
## 18 8 34 5.04
## 19 8 36 5.18
## 20 8 38 4.92
## 21 8 40 7.27
## 22 8 42 2.67
## 23 8 44 2.69
## 24 8 46 2.73
## 25 8 48 4.47
## 26 8 50 4.78
## 27 8 52 6.14
## 28 8 54 3.95
## 29 8 56 3.26
## 30 8 58 3.84
## 31 8 60 2.89
## 32 8 62 4.72
## 33 8 64 5.12
## 34 8 66 6.35
## 35 8 68 6.60
## 36 8 70 3.14
## 37 8 72 2.38
## 38 8 74 4.10
## 39 8 76 5.14
## 40 8 78 5.17
## 41 8 80 5.45
## 42 8 82 6.17
## 43 8 84 3.06
## 44 8 86 2.29
## 45 8 88 5.00
## 46 8 90 4.65
## 47 8 92 5.74
## 48 8 94 6.71
## 49 8 96 7.24
## 50 8 98 3.01
## 51 8 100 2.88
## 52 8 102 2.21
## 53 8 104 4.11
## 54 8 106 5.75
## 55 8 108 6.12
## 56 8 110 5.84
## 57 8 112 4.20
## 58 8 114 2.75
## 59 8 116 2.89
## 60 8 118 3.87
## 61 8 120 6.46
## 62 8 122 8.47
## 63 8 124 8.92
## 64 8 126 5.91
## 65 8 128 2.30
## 66 8 130 1.92
## 67 8 132 3.31
## 68 8 134 4.21
## 69 8 136 8.78
## 70 8 138 7.57
## 71 8 140 5.93
## 72 8 142 2.20
## 73 8 144 2.14
## 74 8 146 3.48
## 75 8 148 5.32
## 76 8 150 7.33
## 77 8 152 10.33
## 78 8 154 6.02
## 79 8 156 4.71
## 80 8 158 1.97
## 81 8 160 2.95
## 82 8 162 5.22
## 83 8 164 7.70
## 84 8 166 7.44
## 85 8 168 5.24
## 86 8 170 3.41
## 87 8 172 1.79
## 88 8 174 3.12
## 89 8 176 4.29
## 90 8 178 6.09
## 91 8 180 6.07
## 92 8 182 7.25
## 93 8 184 2.86
## 94 8 186 2.12
## 95 8 188 2.53
## 96 8 190 4.62
## 97 8 192 8.01
## 98 8 194 10.43
## 99 8 196 8.74
## 100 8 198 4.53
## 101 8 200 2.00
## 102 8 202 2.65
## 103 8 204 3.92
## 104 8 206 6.30
## 105 8 208 11.45
## 106 8 210 10.42
## 107 8 212 7.81
## 108 8 214 2.32
## 109 8 216 2.18
## 110 8 218 4.04
## 111 8 220 6.52
## 112 8 222 7.75
## 113 8 224 13.57
## 114 8 226 10.01
## 115 8 228 4.96
## 116 8 230 1.88
## 117 8 232 3.07
## 118 8 234 5.92
## 119 8 236 8.53
## 120 8 238 11.77
## 121 8 240 11.39
## 122 8 242 7.77
## 123 8 244 2.02
## 124 8 246 2.33
## 125 8 248 3.94
## 126 8 250 7.17
## 127 8 252 10.64
## 128 8 254 13.05
## 129 8 256 10.73
## 130 8 258 4.63
## 131 8 260 2.47
## 132 8 262 2.46
## 133 8 264 5.33
## 134 8 266 8.67
## 135 8 268 13.74
## 136 8 270 13.11
## 137 8 272 8.52
## 138 8 274 3.03
## 139 8 276 2.64
## 140 8 278 4.12
## 141 8 280 7.45
## 142 8 282 13.27
## 143 8 284 14.70
## 144 8 286 10.33
## 145 8 288 4.25
## 146 8 290 2.56
## 147 8 292 2.89
## 148 8 294 5.60
## 149 8 296 12.46

# in this example we reverse the time series, so that the figures are comparable
reversed_df <- male.bowhead.testosterone[rev(rownames(male.bowhead.testosterone)), ]
# hormLong requires dates for the samples, so we can convert our measurements to a date
# NOTE - these dates are not representative of the actual dates of these samples
# however if the actual dates are know, or estimated dates, you could use those.
reversed_df$date <- as.Date(paste0(2009, "-01-01")) + (reversed_df$Dist_from_base_cm -1)
# this sets the data into a structure that hormLong likes to use
reversed_df <- hormDate(data = reversed_df, date_var = 'date', date_order = "ymd")

## Loading required package: lubridate

##
## Attaching package: 'lubridate'

## The following objects are masked from 'package:base':
##
## date, intersect, setdiff, union

# reverts the data back into a data frame
reversed_df <- as.data.frame(reversed_df)
# This will run the iterative process to determine baseline and identify the peaks
result = hormBaseline(data = reversed_df,
 by_var = 'Individual', # grouping variable
 conc_var = 'Testosterone_ng.g', # hormone concentration
 time_var = 'date', # date/time variable
 criteria = 2) # standard deviations used to calculate baseline

##
## [1] "*--- Iteration History ----*"
## [1] "Iteration = 1 : total removed is 10"
## [1] "Iteration = 2 : total removed is 7"
## [1] "Iteration = 3 : total removed is 7"
## [1] "Iteration = 4 : total removed is 2"
## [1] "Iteration = 5 : total removed is 3"
## [1] "Iteration = 6 : total removed is 3"
## [1] "Iteration = 7 : total removed is 5"
## [1] "Iteration = 8 : total removed is 0"
##
## *********
## Note: table saved at:
## C:/Users/jreed/OneDrive - Ursinus College/Post-Doc/TIme Series Analysis paper/Code/TSA_SICB_2026/hormBaseData.csv
## *****

# this will create a figure and save it to your R folder as a PDF.
hormArea(result, lower_bound='peak')

## Loading required package: zoo

## Warning: package 'zoo' was built under R version 4.4.3

##
## Attaching package: 'zoo'

## The following objects are masked from 'package:base':
##
## as.Date, as.Date.numeric

##
## *********
## Note: plots are saved at:
## C:/Users/jreed/OneDrive - Ursinus College/Post-Doc/TIme Series Analysis paper/Code/TSA_SICB_2026/hormArea.pdf
## *****
##
##
## *********
## Note: table saved at:
## C:/Users/jreed/OneDrive - Ursinus College/Post-Doc/TIme Series Analysis paper/Code/TSA_SICB_2026/hormAUCtable.csv
## *****

## scorepeak

We will now show you how to identify peaks using the scorepeak function

library(scorepeak) # loads the scorepeak package
library(tidyverse) # loads tidyverse package - which we will use alongside scorepeak

## Warning: package 'ggplot2' was built under R version 4.4.3

## Warning: package 'purrr' was built under R version 4.4.3

## Warning: package 'stringr' was built under R version 4.4.3

## ── Attaching core tidyverse packages ──────────────────────── tidyverse 2.0.0 ──
## ✔ dplyr 1.1.4 ✔ readr 2.1.5
## ✔ forcats 1.0.0 ✔ stringr 1.5.2
## ✔ ggplot2 4.0.0 ✔ tibble 3.2.1
## ✔ purrr 1.2.0 ✔ tidyr 1.3.1
## ── Conflicts ────────────────────────────────────────── tidyverse_conflicts() ──
## ✖ dplyr::filter() masks stats::filter()
## ✖ dplyr::lag() masks stats::lag()
## ℹ Use the conflicted package (<http://conflicted.r-lib.org/>) to force all conflicts to become errors

# again we will be using the testosterone data to show this
male.bowhead.testosterone # data set

## Individual Dist_from_base_cm Testosterone_ng.g
## 1 8 0 1.94
## 2 8 2 2.60
## 3 8 4 3.12
## 4 8 6 3.54
## 5 8 8 3.88
## 6 8 10 5.51
## 7 8 12 4.17
## 8 8 14 1.96
## 9 8 16 2.28
## 10 8 18 3.02
## 11 8 20 3.80
## 12 8 22 3.69
## 13 8 24 5.27
## 14 8 26 4.27
## 15 8 28 3.40
## 16 8 30 2.26
## 17 8 32 2.69
## 18 8 34 5.04
## 19 8 36 5.18
## 20 8 38 4.92
## 21 8 40 7.27
## 22 8 42 2.67
## 23 8 44 2.69
## 24 8 46 2.73
## 25 8 48 4.47
## 26 8 50 4.78
## 27 8 52 6.14
## 28 8 54 3.95
## 29 8 56 3.26
## 30 8 58 3.84
## 31 8 60 2.89
## 32 8 62 4.72
## 33 8 64 5.12
## 34 8 66 6.35
## 35 8 68 6.60
## 36 8 70 3.14
## 37 8 72 2.38
## 38 8 74 4.10
## 39 8 76 5.14
## 40 8 78 5.17
## 41 8 80 5.45
## 42 8 82 6.17
## 43 8 84 3.06
## 44 8 86 2.29
## 45 8 88 5.00
## 46 8 90 4.65
## 47 8 92 5.74
## 48 8 94 6.71
## 49 8 96 7.24
## 50 8 98 3.01
## 51 8 100 2.88
## 52 8 102 2.21
## 53 8 104 4.11
## 54 8 106 5.75
## 55 8 108 6.12
## 56 8 110 5.84
## 57 8 112 4.20
## 58 8 114 2.75
## 59 8 116 2.89
## 60 8 118 3.87
## 61 8 120 6.46
## 62 8 122 8.47
## 63 8 124 8.92
## 64 8 126 5.91
## 65 8 128 2.30
## 66 8 130 1.92
## 67 8 132 3.31
## 68 8 134 4.21
## 69 8 136 8.78
## 70 8 138 7.57
## 71 8 140 5.93
## 72 8 142 2.20
## 73 8 144 2.14
## 74 8 146 3.48
## 75 8 148 5.32
## 76 8 150 7.33
## 77 8 152 10.33
## 78 8 154 6.02
## 79 8 156 4.71
## 80 8 158 1.97
## 81 8 160 2.95
## 82 8 162 5.22
## 83 8 164 7.70
## 84 8 166 7.44
## 85 8 168 5.24
## 86 8 170 3.41
## 87 8 172 1.79
## 88 8 174 3.12
## 89 8 176 4.29
## 90 8 178 6.09
## 91 8 180 6.07
## 92 8 182 7.25
## 93 8 184 2.86
## 94 8 186 2.12
## 95 8 188 2.53
## 96 8 190 4.62
## 97 8 192 8.01
## 98 8 194 10.43
## 99 8 196 8.74
## 100 8 198 4.53
## 101 8 200 2.00
## 102 8 202 2.65
## 103 8 204 3.92
## 104 8 206 6.30
## 105 8 208 11.45
## 106 8 210 10.42
## 107 8 212 7.81
## 108 8 214 2.32
## 109 8 216 2.18
## 110 8 218 4.04
## 111 8 220 6.52
## 112 8 222 7.75
## 113 8 224 13.57
## 114 8 226 10.01
## 115 8 228 4.96
## 116 8 230 1.88
## 117 8 232 3.07
## 118 8 234 5.92
## 119 8 236 8.53
## 120 8 238 11.77
## 121 8 240 11.39
## 122 8 242 7.77
## 123 8 244 2.02
## 124 8 246 2.33
## 125 8 248 3.94
## 126 8 250 7.17
## 127 8 252 10.64
## 128 8 254 13.05
## 129 8 256 10.73
## 130 8 258 4.63
## 131 8 260 2.47
## 132 8 262 2.46
## 133 8 264 5.33
## 134 8 266 8.67
## 135 8 268 13.74
## 136 8 270 13.11
## 137 8 272 8.52
## 138 8 274 3.03
## 139 8 276 2.64
## 140 8 278 4.12
## 141 8 280 7.45
## 142 8 282 13.27
## 143 8 284 14.70
## 144 8 286 10.33
## 145 8 288 4.25
## 146 8 290 2.56
## 147 8 292 2.89
## 148 8 294 5.60
## 149 8 296 12.46

# we will use a pipeline through tidyverse so that all functions are done simultaneously
# this first line calls the data set and assigns it a new name (testo)
Testo <- male.bowhead.testosterone %>%
 # this second line uses the `detect_localmaxima` argument to find the peaks in the data
 # 5 is set as the window to identify these peaks, looking at each data point and comparing
 # it to its two neighbours before and after. You an change this window depending on the
 # frequency of your peaks and data, setting too large will fail to identify peaks,
 # and too small will identify too many peaks.
 mutate(local_peaks = detect_localmaxima(male.bowhead.testosterone$`Testosterone_ng.g`, 5),
 # This section uses the `score` argument to score the peaks, this will determine if the
 # identified peak is a true peak or just selected due to noise in the data.
 # again we set the window size to the same value as the first argument
 score = score_type1(male.bowhead.testosterone$`Testosterone_ng.g`, 5)) %>%
 # This section will select the true peaks who were both identified as local peaks
 # and scored as true peaks to be returned in the Testo object.
 mutate(true_peaks = score> 0.03 & local_peaks == T)

# Now we want to identify the location of these peaks, rather than having a True or
# False, to do this we can use an `ifelse` argument:
# - the first part is what we are testing (is the peak true)
# - the second is what we do is that is true (get the location of that peak)
# - third is what happens if false (NA)
Testo$peak <- ifelse(Testo$true_peaks == T, Testo$Dist_from_base_cm, NA)

# now that we have our identified peaks, we can plot them using ggplot
library(ggplot2) # load ggplot
ggplot(Testo, # this is the data we are plotting
 aes(x = Dist_from_base_cm, # our x axis
 y = `Testosterone_ng.g`)) + # our y axis
 geom_point(colour = "slateblue4", size = 2) + # this section we can set the colour
 geom_line(colour = "slateblue4") + # of the lines and points and their size
 geom_vline(aes(xintercept = peak), color = "red") + # this adds vertical lines where
 # our peaks have been identified.
 theme_bw() + # this is the theme of the plot (bw removes unnecessary details)
 labs(title = str_c( 'Testosterone profile'), # this lets us change the title
 x = "Distance from base (cm)", # we can change the x axis label
 y = "ng/g")+ # and the y axis label
 scale_x_reverse() # this argument reverses the x axis scale so that the plot goes

## Warning: Removed 128 rows containing missing values or values outside the scale range
## (`geom_vline()`).


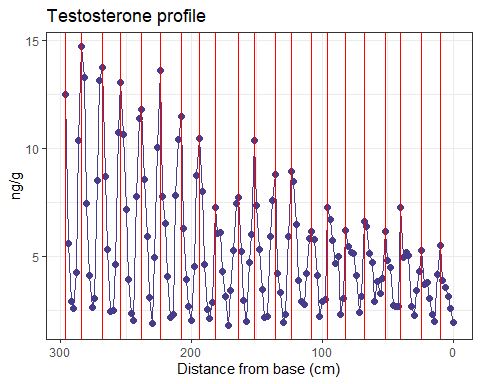


# in chronological order, like the other plots we have presented

# Section.5 Amplitude analysis

To do amplitude analysis we are using the time-varying amplitude from Applied Time Series Analysis for Fisheries and Environmental Sciences by Holmes, Scheuerell and Ward (2021). For this we will be using the MARSS package

library(MARSS) # load the MARSS package

## Warning: package 'MARSS' was built under R version 4.4.3

# first we need to create an object of the length of our data set
TT <- 149 # we have 149 samples from this baleen plate
# next we need to create two covariates for the sin and cosine curves, these are
# both divided by seven, whih is roughly the frequency of seasonal peaks.
cov1 <- sin(2 * pi * (1:TT)/7)
cov2 <- cos(2 * pi * (1:TT)/7)

# next we create an z matrix for our data to be stored
Z <- array(list(1), dim = c(1, 2, TT))
# we then set the vaules for the first two components of the z matrix, again seven
# reflecs the frequency of the seasonal cycles
Z[1, 2, ] <- paste0(sin(2 * pi * (1:TT)/7), " + ", cos(2 * pi *
 (1:TT)/7), "*beta")

# we can then set our model list, these are the arguments that the model will use to fit
mod.list <- list(U = "zero", Q = "diagonal and unequal", Z = Z,
 A = "zero")

# now we can fit the model, we specified method="BFGS" to help with model convergence
# and we increased the maximum number of iterations to 8000. These arguments can be
# changed depending on the complexity and size of your data.
fit <- MARSS(reversed_ts, method="BFGS", control = list(maxit = 8000), model = mod.list, inits = list(x0 = matrix(0,
 2, 1)))

## Success! Converged in 63 iterations.
## Function MARSSkfas used for likelihood calculation.
##
## MARSS fit is
## Estimation method: BFGS
## Estimation converged in 63 iterations.
## Log-likelihood: -316.3152
## AIC: 644.6303 AICc: 645.2219
##
## Estimate
## Z.beta 2.47e+00
## R.R 5.20e-07
## Q.(X1,X1) 3.59e+00
## Q.(X2,X2) 2.06e-02
## x0.X1 7.55e+00
## x0.X2 2.11e+00
## Initial states (x0) defined at t=0
##
## Standard errors have not been calculated.
## Use MARSSparamCIs to compute CIs and bias estimates.

# We can now extract the parameters of interest from the model and combine them into
# a single data frame.
df <- (data.frame(t=1:TT, value=fit$states[2,], type="estimate", var="amplitude scaling"))
df <- rbind(df, data.frame(t=1:TT, value=fit$states[1,], type="estimate", var="xt"))
df <- rbind(df, data.frame(t=1:7, value=cov1[1:7]+coef(fit)$Z[1]*cov2[1:7], type="estimate", var="season"))

# now we can plot these output to see how seasonal amplitude changes in the time series
ggplot(df, aes(x=t, y=value, color=type)) + geom_line() + facet_wrap(~var, scales="free")


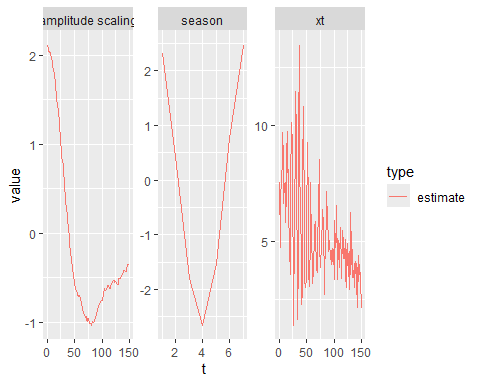


# Section.6 Spectral Analysis

Here we show how to conduct spectral analysis using the spectrum function. This function allows for the estimation of spectral densities of time series data. We will be using the male testosterone data to show this again, with the reveresed time series.

reversed_ts # reversed time series

## [1] 12.46 5.60 2.89 2.56 4.25 10.33 14.70 13.27 7.45 4.12 2.64 3.03
## [13] 8.52 13.11 13.74 8.67 5.33 2.46 2.47 4.63 10.73 13.05 10.64 7.17
## [25] 3.94 2.33 2.02 7.77 11.39 11.77 8.53 5.92 3.07 1.88 4.96 10.01
## [37] 13.57 7.75 6.52 4.04 2.18 2.32 7.81 10.42 11.45 6.30 3.92 2.65
## [49] 2.00 4.53 8.74 10.43 8.01 4.62 2.53 2.12 2.86 7.25 6.07 6.09
## [61] 4.29 3.12 1.79 3.41 5.24 7.44 7.70 5.22 2.95 1.97 4.71 6.02
## [73] 10.33 7.33 5.32 3.48 2.14 2.20 5.93 7.57 8.78 4.21 3.31 1.92
## [85] 2.30 5.91 8.92 8.47 6.46 3.87 2.89 2.75 4.20 5.84 6.12 5.75
## [97] 4.11 2.21 2.88 3.01 7.24 6.71 5.74 4.65 5.00 2.29 3.06 6.17
## [109] 5.45 5.17 5.14 4.10 2.38 3.14 6.60 6.35 5.12 4.72 2.89 3.84
## [121] 3.26 3.95 6.14 4.78 4.47 2.73 2.69 2.67 7.27 4.92 5.18 5.04
## [133] 2.69 2.26 3.40 4.27 5.27 3.69 3.80 3.02 2.28 1.96 4.17 5.51
## [145] 3.88 3.54 3.12 2.60 1.94

spec_ts_data <- ts(reversed_ts, start = 1) # create a new time series without the frequency
detr <- diff(spec_ts_data) # Detrend the data to remove any trend
del<-1 # sampling interval (set as one because that's how its recorded in the time series)
x.spec <- spectrum(detr) # run the spectral analysis on the data and record to an object


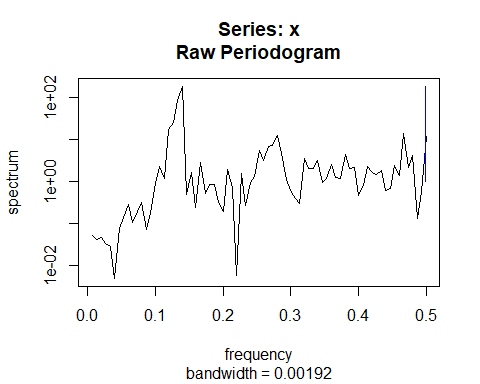


spx <- x.spec$freq/del # recover the frequency component from the spectral analysis
 # and divide by the sampling frequency
spy <- 2*x.spec$spec # recover the spectral density from the analysis. We multiply
 # this by two so the area under the periodogram equals the variance
 # of the time series.
plot(spy~spx,xlab="frequency",ylab="spectral density",type="l") # we can now plot the


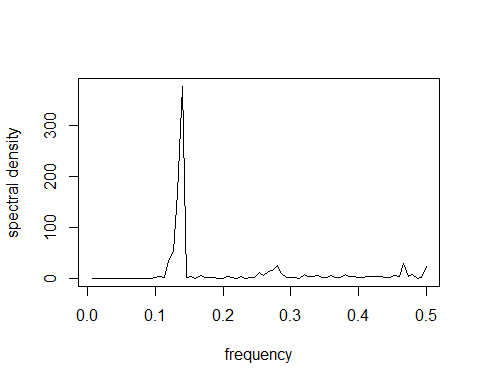


# periodogram to see there the spikes are occurring.
# to calculate the frequency from the find the location of the significant spikes
# firstly we find the highest spectral density in the output, ours was at location 21
# (value 537), we then find the 21st value from the frequencies (0.14).
# now we can calculate the frequency, giving us ~14.286.
freq <- (1/0.14)*2
