# Supplementary material for: Time Series Analysis for Physiological and Endocrinological Data: A Practical Guide
Source: Integr Comp Biol. 2026 Jun 18;66:icag092. doi: 10.1093/icb/icag092 (PMC13339088; doi:10.1093/icb/icag092)
Supplement: icag092_Supplemental_Files [file icag092_supplemental_files.zip › icb-2026-0050-File016.docx]

**Supplemental Information - resources**

Time series

- <https://www.abs.gov.au/websitedbs/d3310114.nsf/home/time+series+analysis:+the+basics>
- <https://www.ncbi.nlm.nih.gov/books/NBK543532/>

ARIMA – including autoregressive and moving average models

- [https://www.geeksforgeeks.org/understanding-partial-autocorrelation-functions-pacf-in-time-series-data/](https://www.google.com/url?q=https://www.geeksforgeeks.org/understanding-partial-autocorrelation-functions-pacf-in-time-series-data/&sa=D&source=docs&ust=1754497855744104&usg=AOvVaw2BEEnX4jSc8JLR_NGYULSk)
- [https://rpubs.com/JSHAH/481706](https://www.google.com/url?q=https://rpubs.com/JSHAH/481706&sa=D&source=docs&ust=1754497855736648&usg=AOvVaw0MXZExJxsXYCuTPfDPvyM3) - This page is a good resource for fitting AR and MA models with background and examples.
- [https://people.duke.edu/~rnau/411arim.htm](https://www.google.com/url?q=https://people.duke.edu/~rnau/411arim.htm&sa=D&source=docs&ust=1754497855742406&usg=AOvVaw3-5afbgVrWhEmGM2lKkjPB)
- [https://otexts.com/fpp2/seasonal-arima.html](https://www.google.com/url?q=https://otexts.com/fpp2/seasonal-arima.html&sa=D&source=docs&ust=1754497855732891&usg=AOvVaw30xKpq_4hxsjybmyYSAcTL)
- [https://www.ibm.com/think/topics/arima-model](https://www.google.com/url?q=https://www.ibm.com/think/topics/arima-model&sa=D&source=docs&ust=1754497855733325&usg=AOvVaw3WH287o3pC2WCrvNxSjcgF)
- [https://people.duke.edu/~rnau/seasarim.htm](https://www.google.com/url?q=https://people.duke.edu/~rnau/seasarim.htm&sa=D&source=docs&ust=1754497855742984&usg=AOvVaw2vLcosq6VNthZvjqIbE8Hd)
- [https://online.stat.psu.edu/stat510/Lesson04](https://www.google.com/url?q=https://online.stat.psu.edu/stat510/Lesson04&sa=D&source=docs&ust=1754497855733904&usg=AOvVaw3JnST_4FxGYWxo0egRdIHu)

Cross-correlation function

- [https://online.stat.psu.edu/stat510/lesson/8/8.2](https://www.google.com/url?q=https://online.stat.psu.edu/stat510/lesson/8/8.2&sa=D&source=docs&ust=1754497855735373&usg=AOvVaw2dJcahel6s0Mq16R9GRX_K)
- <https://online.stat.psu.edu/stat510/lesson/9/9.1>

Spectral Analysis

- <https://www.rdocumentation.org/packages/stats/versions/3.6.2/topics/spectrum>
- https://online.stat.psu.edu/stat510/Lesson06
- <https://online.stat.psu.edu/stat510/Lesson12>
- https://web.stanford.edu/class/earthsys214/notes/series.html
- <https://share.google/svw9nsnD43zGhJXkw>
- Chapter 4 in *Time Series Analysis and its Applications: with R examples* by Shumway, R.H. and Stoffer, D.S. (2017) 4^th^ edition.
- Chapter 13 in *Time Series Analysis: with applications in R* by Cryer, J.D., and Chan, KS. (2008).

Peak analysis

- <https://hormlong.weebly.com/>
- <https://github.com/bfanson/hormLong/tree/master/vignettes>
- https://cran.r-project.org/web/packages/scorepeak/vignettes/intro.html

Amplitude analysis

- <https://atsa-es.github.io/atsa-labs/time-varying-amplitude.html>

Change point models

- https://cran.r-project.org/web/packages/ecp/vignettes/ecp.pdf
- <https://cran.r-project.org/web/packages/changepoint/changepoint.pdf>
- <https://cran.r-project.org/web/packages/changepoint/index.html>
- <https://stat153.berkeley.edu/spring-2025/lab5>

Autoregressive Conditionally Heteroscedastic (ARCH) models

- <https://online.stat.psu.edu/stat510/Lesson11>
- <https://cran.r-project.org/web/packages/rugarch/vignettes/Introduction_to_the_rugarch_package.pdf>
- https://online.stat.psu.edu/stat510/Lesson11
- <https://bookdown.org/ccolonescu/RPoE4/time-varying-volatility-and-arch-models.html>
